# MicroRNA-874 targets phosphomevalonate kinase and inhibits cancer cell growth via the mevalonate pathway

**DOI:** 10.1038/s41598-022-23205-w

**Published:** 2022-11-02

**Authors:** Alimasi Aersilan, Naoko Hashimoto, Kazuyuki Yamagata, Masataka Yokoyama, Akitoshi Nakayama, Xiaoyan Shi, Hidekazu Nagano, Ikki Sakuma, Nijiro Nohata, Takashi Kinoshita, Naohiko Seki, Bahityar Rahmutulla, Atsushi Kaneda, Siti Nurul Zhahara, Yingbo Gong, Motoi Nishimura, Shoichiro Kawauchi, Eiryo Kawakami, Tomoaki Tanaka

**Affiliations:** 1 Department of Molecular Diagnosis, Graduate School of Medicine, Chiba University, 1-8-1 Inohana, Chuo-Ku, Chiba, Chiba 260-8677 Japan; 2 Department of Functional Genomics, Graduate School of Medicine, Chiba University, Chiba, Japan; 3 Department of Molecular Oncology, Graduate School of Medicine, Chiba University, Chiba, Japan; 4 Division of Laboratory Medicine, Clinical Genetics and Proteomics, Chiba University Hospital, Chiba, Japan; 5 Advanced Data Science Project (ADSP), RIKEN, Kanagawa, Japan; 6 Department of Artificial Intelligence Medicine, Graduate School of Medicine, Chiba University, Chiba, Japan; 7 Research Institute of Disaster Medicine, Chiba University, Chiba, Japan; 8 Present Address: MSD K.K., Tokyo, Japan

**Keywords:** Breast cancer, Apoptosis

## Abstract

The microRNA (miR) *miR-874*, a potential tumour suppressor, causes cell death via target gene suppression in various cancer types. Mevalonate pathway inhibition also causes cell death in breast cancer. However, the relationship between the mevalonate pathway and *miR-874*-induced apoptosis or its association with the tumour suppressor p53 has not been elucidated. We identified phosphomevalonate kinase (PMVK), a key mevalonate pathway enzyme, and sterol regulatory element-binding factor 2 (SREBF2), the master cholesterol biosynthesis regulator, as direct *miR‑874* targets. Next-generation sequencing analysis revealed a significant *miR-874-*mediated downregulation of *PMVK* and *SREBF2* gene expression and p53 pathway enrichment. Luciferase reporter assays showed that *miR-874* directly regulated *PMVK* and *SREBF2*. *miR-874*-induced apoptosis was p53 dependent, and single-cell RNA sequencing analysis demonstrated that *miR-874* transfection resulted in apoptosis and p53 pathway activation. Downregulation of *PMVK* expression also caused cell cycle arrest and p53 pathway activation, which was rescued by geranylgeranyl pyrophosphate (GGPP) supplementation. Analysis of The Cancer Genome Atlas (TCGA) database indicated a negative correlation between *miR-874* and *PMVK* expression and between *miR-874* and *SREBF2* expression. These findings suggest that *miR-874* suppresses the mevalonate pathway by targeting *SREBF2* and *PMVK,* resulting in GGPP depletion, which activates the p53 pathway and promotes cycle arrest or apoptosis.

## Introduction

Breast cancer is among the most common cancers worldwide. Surgical procedures and recent advances in endocrine therapy, chemotherapy, radiotherapy, and targeted molecular therapies have improved breast cancer treatment efficacy. However, breast cancer remains a leading cause of cancer-related deaths among women globally^1^, and multidisciplinary approaches demonstrate limited efficacy in some patients with metastatic breast cancer, requiring the development of novel therapeutic approaches.

Cancer cells undergo metabolic reprogramming, and recent studies have reported the upregulation of the mevalonate pathway in cancer cells^2,3^. Statins reduce blood cholesterol levels by inhibiting the rate-limiting enzyme of the mevalonate pathway, hydroxymethylglutaryl-CoA reductase (HMGCR), and statin use in patients with various cancer types has been associated with reduced mortality^4^. Statins suppress cancer cell growth^5,6^ and induce apoptosis in various tumour cells, including breast cancer cells^7,8^, suggesting the involvement of the mevalonate pathway in breast cancer development. Furthermore, gain-of-function mutants of p53 upregulate the mevalonate pathway, whereas wild-type p53 represses mevalonate pathway genes^2,9^.

MicroRNAs (miRNAs) are single-stranded, small, noncoding RNAs 19–25 nucleotides in length that regulate gene expression by binding to mRNAs and promoting mRNA degradation or translation inhibition^10^. miRNAs regulate several biological functions, including the cell cycle, cell differentiation, and apoptosis^10^, and miRNAs that regulate the mevalonate pathway have been identified. A recent study reported that *miR-21* targets *HMGCR* to regulate triglyceride and cholesterol metabolism in hepatocytes^11^. In addition, *miR-125a* directly targets *HMGCR* to suppress vascular smooth muscle cell proliferation^12^.

*miR-874* was identified as the most significantly downregulated miRNA in maxillary sinus squamous cell carcinoma tissues compared with corresponding normal tissues and was found to suppress cancer cell proliferation and invasion^13^. *miR-874* was reported to inhibit proliferation by targeting *cyclin-dependent kinase 9* (*CDK9*) in breast cancer^14^. In addition, several lines of evidence indicate that *miR-874* functions as a tumour suppressor in various cancer types, including breast cancer, by targeting multiple genes, including *protein phosphatase 1 catalytic subunit alpha* (*PPP1CA*), *histone deacetylase 1* (*HDAC1*), *E2F transcription factor 3* (*E2F3*), *signal transducer-activator of transcription 3* (*STAT3*), *matrix metalloproteinase-2* (*MMP-2*), *urokinase-type plasminogen activator* (*uPA*), *aquaporin-3* (*AQP3*), and *ETS proto-oncogene 1* (*ETS1)*^13,15–20^, many of which positively regulate cell proliferation. It was reported that *miR-874* expression was downregulated in breast cancer tissues compared with normal tissue adjacent to tumour tissue^21^, indicating that *miR-874* is significantly downregulated in breast cancer. The authors also reported that the *miR-874* expression level was correlated with the status of the oestrogen receptor, TNM stage and lymph node metastasis. Moreover, patients with high miR-874 expression exhibited a better prognosis. Regarding the expression of *miR-874* compared to normal mammary epithelial cell lines such as MCF10A, Kilinc S et al. compared the expression of miRNAs in MCF10A cells with stable expression of oncogenes with empty vectors. They performed qPCR analysis using a panel of 384 miRNA primers, and among them, the expression level of *miR-874* was decreased by oncogene expression, such as AURKB and HRAS, compared to the MCF10A control^22^. These findings indicate that miR-874 is downregulated in breast cancer and that its expression level has prognostic significance. However, the role of *miR-874* in breast cancer, especially in cancer cell metabolism, has not been fully elucidated. *miR-874* also markedly induces cell death, but its association with the p53 pathway has not been reported.

In the present study, we investigated the molecular mechanism of miR-874-mediated tumour suppression in breast cancer, particularly in the context of cancer metabolism and the p53 pathway. Next-generation sequencing (NGS) analysis revealed that *miR-874* transfection resulted in the downregulation of sterol regulatory element-binding factor 2 (*SREBF2*), a transcription factor and the master cholesterol biosynthesis regulator^23^, and phosphomevalonate kinase (*PMVK*), a mevalonate pathway enzyme, both of which were identified as direct *miR-874* targets*. miR-874* transfection induced p53-dependent apoptosis in MCF-7 cells. *PMVK* suppression caused cell cycle arrest and p53 pathway activation, which was rescued by geranylgeranyl pyrophosphate (GGPP) supplementation, a mevalonate pathway metabolite. Our findings reveal the involvement of the mevalonate pathway in the mechanisms underlying the tumour-suppressive function of *miR-874* and suggest that *miR-874* could be a potent therapeutic target in breast cancer.

## Results

### Identification of the *miR-874*-modulated molecular pathway and putative target genes in breast cancer cells

**Figure 1:**
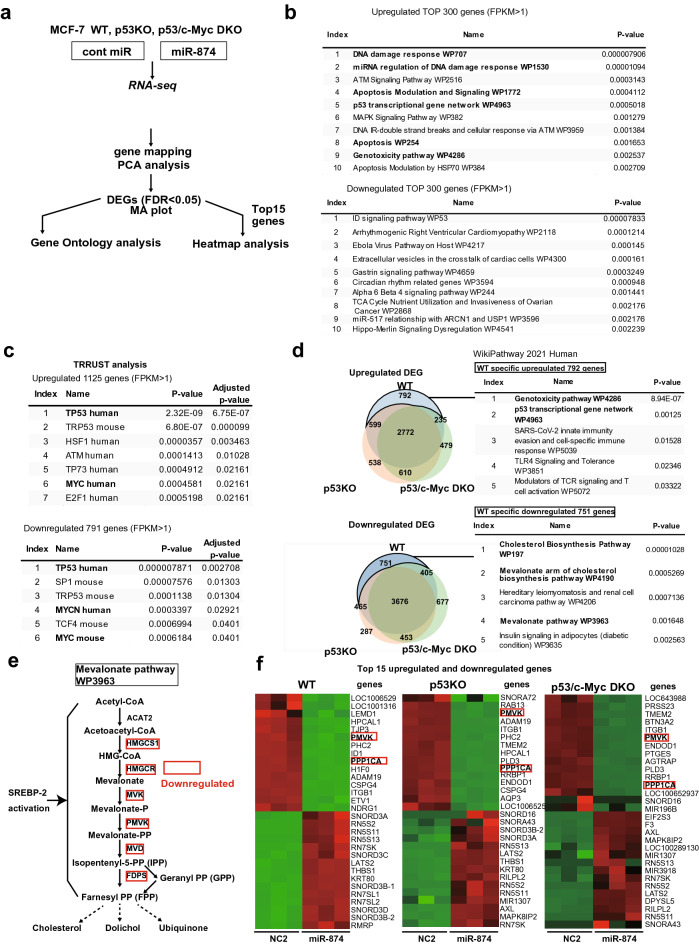
Expression of *miR-874* in MCF-7 cells significantly downregulates the mevalonate pathway. (**a**) Experimental RNA sequencing (RNA-seq) scheme applied to WT, p53 KO and p53/c-Myc DKO MCF-7 cells and subsequent data analysis. (**b**) WikiPathway enrichment analysis of differentially expressed genes (DEGs). Top 10 pathways revealed by WikiPathway 2021 enrichment analysis of DEGs induced by *miR-874* transfection using the top 300 upregulated genes and the top 300 downregulated genes with FPKM > 1 in WT MCF-7 cells. p values (Fisher’s exact test) are presented after the column. Pathways associated with the DNA damage response, apoptosis, and p53 are indicated in bold. (**c**) The top seven and six transcription factors of up- and downregulated mRNA transcripts, respectively, upon *miR-874* overexpression in WT MCF-7 cells were analysed by TRRUST, and p values (Fisher’s exact test) are presented after the column. p53 and Myc are shown in bold. (**d**) Top five pathways of WT MCF-7-specific up- and downregulated DEGs induced by *miR-874* transfection were analysed by WikiPathway 2021. p values (Fisher’s exact test) are presented after the column. Pathways associated with the DNA damage response, p53, and the mevalonate pathway are shown in bold. (**e**) The mevalonate pathway (WP3963) from WikiPathways is shown. Downregulated genes are surrounded by red squares. (**f**) The top 15 upregulated and downregulated genes between WT, p53 KO and p53/c-Myc DKO cells transfected with cont miRNA and *miR-874* are listed in the heatmap using hierarchical clustering. PPP1CA, framed in red, is shown as a positive control because it is a previously reported target gene of *miR-874*.

Several lines of evidence indicate that c-Myc and p53 are essential for the induction of apoptosis or cell cycle arrest^24,25^. To evaluate the tumour-suppressive effects of *miR-874* in breast cancer cells, we employed RNA-seq to compare WT MCF-7 cells transfected with *miR-874* or control miRNA (Fig. 1a). Principal component analysis (PCA) showed the effects of *miR-874* transfection on the gene expression profile represented by the PC1 and PC2 axes (Supplementary Fig. S1a). Differentially expressed genes (DEGs) between WT MCF-7 cells transfected with control miRNA and *miR-874* were identified (Supplementary Fig. S1b). To characterize the genes whose expression was altered by *miR-874*, we focused on upregulated or downregulated genes/pathways in *miR-874*-transfected cells relative to control miRNA-transfected cells (Fig. 1b). As expected, the DNA damage response, apoptosis, and p53 transcriptional gene network were highly upregulated. Furthermore, using TRRUST analysis to search for upstream factors, p53 was the most enriched transcription factor, and Myc was also highly enriched (Fig. 1c). Therefore, we re-analysed RNA-seq for *miR-874* effects on p53 KO and p53/c-Myc DKO cells compared to WT cells. The PCA is shown in Supplementary Fig. S1a. DEGs between p53 KO and p53/c-Myc DKO MCF-7 cells transfected with control miRNA and *miR-874* were also identified (Supplementary Fig. S1b). Therefore, we first focused on the genes that were upregulated by *miR-874* transfection specifically for WT p53. The genotoxic pathway and p53 transcriptional gene network were the most upregulated WT-specific pathways (Fig. 1d). Next, analysis of the DEGs common to the three cell lines revealed an enrichment of the cell cycle rather than apoptosis (Supplementary Fig. S1e). In contrast, the apoptotic pathway was not upregulated in either p53 KO or p53/c-Myc DKO MCF-7 cells (Supplementary Fig. S1e). Since miRNAs exert their function by repressing target mRNAs, to examine the pathways repressed by *miR-874*, we turned to the downregulated genes in WT MCF-7 cells to explore the point of action of *miR-874*. As a result, cholesterol biosynthesis and the mevalonate pathway were found to be highly enriched (Fig. 1d). Expression level analysis of fragments per kilobase of exon per million mapped sequence reads (FPKM) from RNA-seq data confirmed that 5 of 7 genes (*HMGCS*, *HMGCR*, *PMVK, MVD,* and *FDPS*) in the mevalonate pathway and *SREBF1/2,* master regulators of lipid metabolism, were significantly suppressed by *miR-874*, including an 85% reduction in *PMVK* expression (Fig. 1e, Table 1). The effect of *miR-874* on the mevalonate pathway was also examined in p53 KO and p53/c-Myc DKO cells. Similar to WT MCF-7 cells, *miR-874*-transfected p53 KO and p53/c-Myc DKO cells exhibited significantly decreased expression of mevalonate pathway genes (Table 1), according to FPKM RNA-seq data analysis, including more than 85% reduction in *PMVK* expression, although the mevalonate pathway was not identified as a top enriched pathway in pathway enrichment analysis (Supplementary Fig. S1f). Furthermore, heatmap analysis confirmed that *PMVK*, along with *PPP1CA*, one of the previously reported targets of *miR-874*, was among the 15 most significantly downregulated genes in cells transfected with *miR-874* compared with control miRNA, not only in WT but also in p53 KO and p53/c-Myc DKO MCF-7 cells (Fig. 1f).

**Table 1:** Mevalonate pathway and SREBF expression determined by RNA-seq (FPKM).

	p53 WT					p53 KO					p53/c-Myc DKO				
	cont miR		miR-874		cont miR vs miR874; p-value	cont miR		miR-874		cont miR vs miR874; p-value	cont miR		miR-874		cont miR vs miR874; p-value
	Mean	SD	Mean	SD		Mean	SD	Mean	SD		Mean	SD	Mean	SD	
ACAT2	21.269	1.655	18.357	0.755	NS	24.784	0.336	29.303	1.072	p < 0.05	47.291	0.651	57.568	0.261	p < 0.0001
HMGCS1	34.154	1.054	20.724	0.396	p < 0.0001	43.010	0.903	33.410	1.128	p < 0.0001	59.204	1.118	47.104	1.705	p < 0.0001
HMGCR	44.456	1.513	24.433	1.217	p < 0.0001	51.045	1.013	35.925	1.165	p < 0.0001	56.508	0.504	39.850	2.389	p < 0.0001
MVK	10.999	0.622	7.289	0.555	NS	14.299	0.725	13.447	0.805	NS	14.187	0.299	14.514	0.978	NS
PMVK	21.786	1.308	3.365	0.390	p < 0.0001	20.618	0.320	2.754	0.254	p < 0.0001	22.860	0.751	3.282	0.223	p < 0.0001
MVD	32.189	2.904	24.542	1.183	p < 0.0001	34.793	0.816	31.985	2.444	NS	41.436	1.948	44.184	0.448	NS
FDPS	97.056	1.816	77.987	2.291	p < 0.0001	106.200	2.042	102.255	4.402	NS	98.560	1.890	111.743	1.590	p < 0.0001
SREBF1	47.364	1.001	28.755	0.508	p < 0.0001	53.770	3.026	40.695	1.305	p < 0.0001	36.279	0.535	21.323	0.832	p < 0.0001
SREBF2	52.607	1.612	13.998	1.126	p < 0.0001	57.307	1.154	15.114	0.464	p < 0.0001	62.681	1.116	15.873	0.904	p < 0.0001

These results suggest that the tumour-suppressive function of *miR-874* is associated with apoptosis via the p53 and c-Myc signalling pathways. Moreover, our analysis of genes downregulated by *miR-874* transfection indicates that *miR-874* represses genes involved in multiple steps of the mevalonate pathway, most notably *PMVK*, in a p53-independent manner. These results suggest that *PMVK* may be a *miR-874* target.

### *miR-874* directly targets both *PMVK* and *SREBF2,* inhibiting their expression

**Figure 2:**
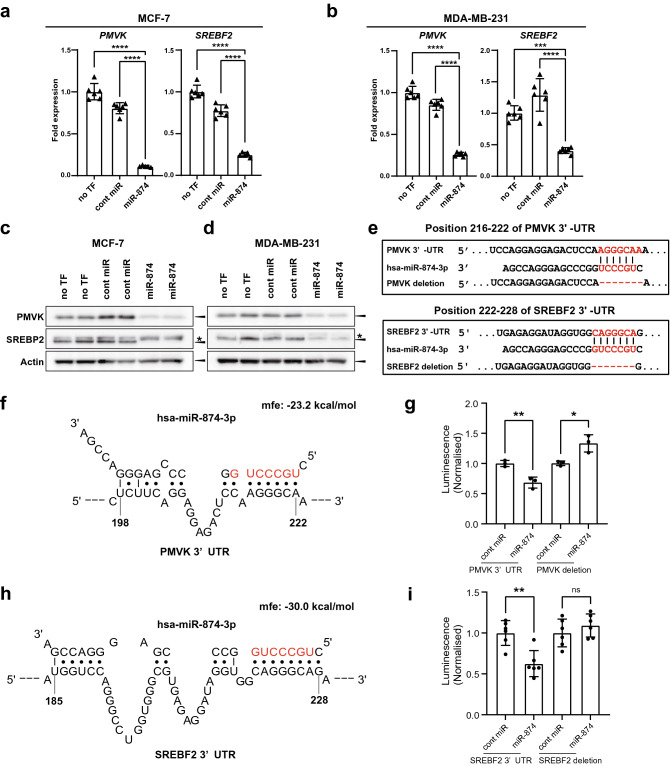
Identification of PMVK as a novel *miR-874* target in breast cancer cells. (**a**,**b**) Quantitative reverse-transcription polymerase chain reaction (qRT-PCR) assays were used to analyse the effects of *miR-874* on *PMVK* mRNA and *SREBF2* mRNA in MCF-7 and MDA-MB-231 cells. Data are presented as the mean ± SD. ***p < 0.001, ****p < 0.0001 (one-way ANOVA). (**c**,**d**) Western blotting for PMVK, SREBP2, and actin in MCF-7 and MDA-MB-231 cells transfected with cont miRNA or *miR-874* for 72 h. Triangles indicate specific bands, and asterisks indicate nonspecific bands. (**e**) Bioinformatic analysis using TargetScan predicted that *miR-874* might target both the *PMVK* 3′-UTR and the *SREBF2* 3’-UTR. (**f**) Putative *miR-874* sites in the 3′-UTR of *PMVK,* as determined by the STarMir program. (**g**) p53/c-Myc double-knockout (DKO) MCF-7 cells were transfected with the *PMVK* 3′-UTR in a vector construct and *miR-874* or control (cont) miRNA to analyse the effects on luciferase activity in the psiCHECK-2-PMVK-3′-UTR reporter. n = 3. Data are presented as the mean ± SD. *p < 0.05, **p < 0.01 (Student’s t test). (**h**) Putative *miR-874* sites in the 3′-UTR of *SREBF2,* as determined by the STarMir program. (**i**) p53/c-Myc DKO MCF-7 cells were transfected with the *SREBF2* 3′-UTR in a vector construct and *miR-874* or cont miRNA to analyse the effects on luciferase activity in the psiCHECK-2-SREBF2-3′-UTR reporter. n = 6. Data are presented as the mean ± SD. **p < 0.01 (Student’s t test).

The RNA-seq results showed that *miR-874* acts on multiple components of the mevalonate pathway, including *PMVK*, which is necessary for the maintenance of cell cycle progression^26^ (Fig. 1e, Table 1). However, even if *PMVK* is a direct target, it alone does not explain the repression of genes in multiple steps of the mevalonate pathway. In our RNA-seq analysis, we found that the expression of *SREBF2* was also downregulated by *miR-874* transfection. To validate the RNA-seq results, we assessed whether *miR-874* affects endogenous *PMVK* and *SREBF2* expression in breast cancer cell lines. MCF-7 is an oestrogen receptor (ER)-positive and progesterone receptor (PgR)-positive breast cancer cell line that expresses WT p53^27^, whereas MDA-MB-231 is a triple-negative breast cancer cell line that expresses mutant p53 (R280K), which promotes tumour progression^28^. *miR-874* overexpression markedly suppressed *PMVK* and *SREBF2* mRNA expression in both MCF-7 and MDA-MB-231 cells (Fig. 2a,b), and *HMGCR* was also downregulated in both MCF-7 and MDA-MB-231 cells (Supplementary Fig. S2), despite a lack of predicted *miR-874* target sites in the 3′-UTR of *HMGCR* according to the TargetScan database. The protein expression levels of PMVK and SREBP2 also decreased following transfection with *miR-874* compared with control miRNA in both cell lines (Fig. 2c,d). To explore the mechanism of *PMVK* and *SREBF2* downregulation by *miR-874*, we employed the TargetScan database to predict *miR-874* target genes. Putative *miR-874* target sites were located in positions 216–222 of the *PMVK* 3′-UTR and in positions 222–228 of the *SREBF2* 3′-UTR (Fig. 2e). The genes that were downregulated also contained predicted seed sites for *miR-874* and are shown in Supplementary Table S1. STarMir predicted that the seed sequence for *PMVK* and *miR-874* binding had a minimum free energy of − 23.2 kcal/mol, suggesting that *miR-874* might form a stable structure with *PMVK* and *SREBF2,* respectively (Fig. 2f,h). To investigate whether *miR-874* targets *PMVK* and *SREBF2* directly, a reporter assay was performed by constructing psiCHECK2 vectors containing an intact *PMVK* 3′-UTR or mutant *PMVK* 3’-UTR lacking the *miR-874* binding site. Similarly, WT or miR-874 binding site-deleted *SREBF2* 3′-UTR fragments were cloned into the psiCHECK2 vector downstream of the Renilla luciferase reporter gene. p53/c-Myc DKO MCF-7 cells were cotransfected with *miR-874* and the reporter vectors. Compared with the control miRNA, *miR-874* reduced the luciferase activities of the *PMVK* 3′-UTR and *SREBF2* 3′-UTR WT reporters, but it had no effect on the constructs lacking the binding site (Fig. 2g,i). Deletion of positions 216–222 of the 3′-UTR of PMVK increased the luminescence (Fig. 2g, right). These results suggest that *PMVK* and *SREBF2* are direct targets of *miR-874*.

### *miR-874* activates p53 and induces apoptotic cell death and tumour cell growth inhibition

**Figure 3:**
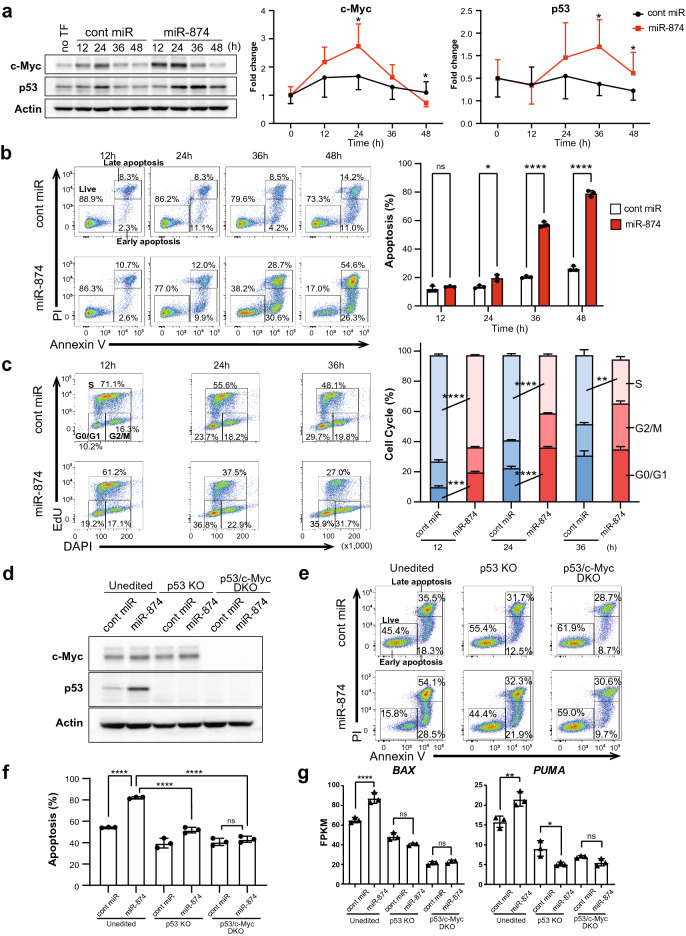
*miR-874* induces p53-mediated cell death and suppresses cell proliferation in a p53-dependent manner. (**a**) Changes in the expression levels of c-Myc and p53 with *miR-874* transfection over time. MCF-7 cells were transfected with control (cont) miRNA or *miR-874*. The cells were harvested at the indicated time points and subjected to western blotting. Representative western blot images are shown (left). A line chart showing changes in the expression levels of c-Myc and p53 (right). n = 5, mean ± SD, *p < 0.05 (Student’s t test). See also Supplementary Fig. S3. (**b**) Annexin V time-course study. MCF-7 cells were transfected with cont miRNA or *miR-874*. The cells were harvested at the indicated time points and subjected to an annexin V-633/propidium iodide (PI) double-staining assay. Representative images are shown (left). The percentages of annexin V-positive cells/PI-negative (lower right, early apoptosis) and annexin V-positive/PI-positive cells (upper right, late apoptosis) were quantified (right). n = 3 per group, mean ± SD, *p < 0.05, ****p < 0.0001 (Student’s t test). (**c**) EdU time-course study. MCF-7 cells were transfected with cont *miRNA* or *miR-874*. The cells were harvested at the indicated time points and subjected to the EdU assay using flow cytometry analysis. Representative images are shown (left). The proportions of cells in the S phase, G0/G1 phase, and G2/M phase were quantitated and plotted (right). n = 3, mean ± SD, **p < 0.01, ***p < 0.001, ****p < 0.0001 (Student’s t test). (**d**) WT, p53 knockout (KO), and p53/c-Myc double-knockout (DKO) MCF-7 cells were transfected with either cont miRNA or *miR-874*. After 40 h, the cells were harvested and subjected to western blotting. (**e**) WT, p53 KO, and p53/c-Myc DKO MCF-7 cells were transfected with either cont miRNA or *miR-874*. After 48 h, the cells were harvested and subjected to annexin V-633/PI double staining. Representative images are shown. (**f**) The percentages of annexin V-633-positive/PI-negative cells and annexin V-633-positive/PI-positive cells were quantified. n = 3, mean ± SD, ****p < 0.0001 (one-way ANOVA). (**g**) FPKM values for *BAX* and *PUMA* from RNA-seq analysis for WT, p53 KO, and p53/c-Myc DKO MCF-7 cells (n = 3 per group, *p < 0.05, **p < 0.01, ***p < 0.001, ****p < 0.0001, one-way ANOVA).

To assess the tumour-suppressive functions of *miR-874* in breast cancer cells, MCF-7 cells were transfected with control miRNA or *miR-874* mimic. The RNA-seq results showed the upregulation of p53 and c-Myc pathway components following *miR-874* mimic transfection; therefore, we investigated the effects of *miR-874* on p53 and c-Myc expression. First, we examined changes in c-Myc and p53 protein expression over time after *miR-874* transfection, which revealed that c-Myc expression peaked 24 h after *miR-874* transfection before slowly decreasing. p53 expression increased gradually, peaking 36 h after transfection (Fig. 3a, Supplementary Fig. S3). These results suggest that *miR-874* activates c-Myc and p53 sequentially, resulting in apoptosis. To investigate the time course of *miR-*874-induced apoptosis, we performed an annexin V apoptosis assay. Consistent with the p53 activation results, apoptosis was initiated 36 h after *miR-874* transfection and increased at 48 h (Fig. 3b). To investigate the effects of *miR-874* on cell cycle regulation over time, we performed an EdU assay in *miR-874*-transfected MCF-7 cells. A decrease in the proportion of S phase cells and the arrest of cell cycles in the G1 phase were observed as early as 12 h after transfection (Fig. 3c). These data corroborate the findings that *miR-874* activates the c-Myc and p53 signalling pathways. The importance of p53 and c-Myc status on the effects of *miR-874* transfection in WT, p53 KO, and p53/c-Myc DKO MCF-7 cells was examined. Both p53 and p53/c-Myc KO cell generation were confirmed to be successful. p53 upregulation was observed in WT MCF-7 cells transfected with *miR-874* but not in p53 KO or p53/c-Myc DKO MCF-7 cells transfected with *miR-874* (Fig. 3d). Consistently, although *miR-874* overexpression caused a significant increase in the fraction of annexin V-positive p53 WT MCF-7 cells, the apoptosis rate after *miR-874* transfection increased in p53 KO cells but was lower than that in p53 WT cells. Furthermore, the transfection of *miR-874* did not increase the fraction of apoptotic cells relative to control miR transfection in p53/c-Myc DKO cells (Fig. 3e,f). Correspondingly, the RNA-seq results revealed increased expression of *BAX* and *PUMA* in WT MCF-7 cells but not in p53 KO or p53/c-Myc DKO MCF-7 cells (Fig. 3g). These findings suggest that apoptosis induction by *miR-874* is dependent on p53 and c-Myc. We also examined the effects of *miR-874* on apoptosis and cell cycle regulation in MDA-MB-231 cells containing the p53 R280K missense mutation, which promotes tumour progression and metastasis. Annexin V assays showed no induction of apoptotic cell death in *miR-874*-transfected MDA-MB-231 cells (Supplementary Fig. S4a,b). In contrast, cell growth was suppressed in *miR-874*-transfected MDA-MB-231 cells compared with control miRNA-transfected cells (Supplementary Fig. S4c). An EdU assay was performed to analyse the cell cycle by flow cytometry, showing that compared with control miRNA-transfected cells, *miR-874*-transfected cells displayed increased G1 arrest and decreased progression into the S phase at 48 h (Supplementary Fig. S4d,e). These data indicate that *miR-874* induces p53-dependent apoptosis and cell cycle arrest in a partially p53-independent manner.

### scRNA-seq analysis reveals that *miR-874* upregulates the p53 signalling pathway and downregulates the mevalonate pathway

**Figure 4:**
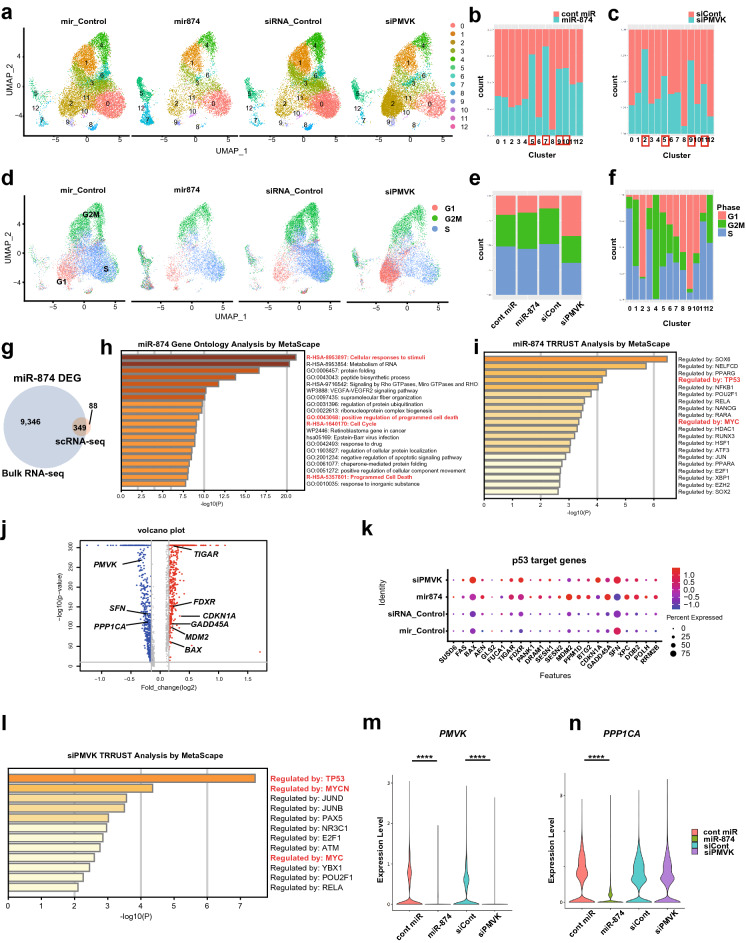
scRNA-seq revealed that *miR-874* downregulates *PMVK* and upregulates p53 target genes in MCF-7 cells. MCF-7 cells were transfected with control (cont) miRNA or *miR-874* for 36 h or control siRNA or siPMVK for 48 h. The viable population of MCF-7 cells was sorted, and each of the four groups was profiled by droplet-based scRNA-seq. (**a**) scRNA-seq data (n = 46,130) across all four groups of treated MCF-7 cells are shown as nonlinear representations of the top 50 principal components. Cells are coloured according to UMAP-based clusters. (**b**,**c**) Proportions of each cluster from miRNA (**b**) or siRNA transfections (**c**). Clusters increased by *miR-874* (**b**) or siPMVK transfection (**c**) are marked with red frames. (**d**) Cells are coloured according to UMAP-based clusters of cell phase, according to treatment. (**e**,**f**) Proportions of the cell phase within each group (**e**) or cluster (**f**). (**g**) Venn diagram showing 349 common differentially expressed genes (DEGs) among 9695 genes identified by bulk RNA-seq and 437 identified by scRNA-seq between cells transfected with cont miRNA and *miR-874*. (**h**) The table shows multiple pathways enriched in 349 common DEGs analysed by Metascape. (**i**) The table shows the prediction of upstream transcription factors among the DEGs by TRRUST analysis in Metascape. (**j**) Volcano plots showing DEGs between cont miRNA and *miR-874.* (**k**) Dot plot showing p53 target genes. (**l**) The table shows the prediction of upstream transcription factors from the DEGs between control siRNA and siPMVK by TRRUST analysis in Metascape. (**m**,**n**) Violin plot of the *miR-874* targets *PMVK* (**m**) and *PPP1CA* (**n**) in each group (****p < 0.0001; Welch’s t test).

*miR-874* affects not only the mevalonate pathway but also cell cycle arrest and p53-mediated apoptosis. To extensively characterize the *miR-874*-mediated cell cycle mechanism and its association with *PMVK* downregulation, we performed scRNA-seq analysis of MCF-7 cells transfected with control miRNA or *miR-874* for 36 h or control siRNA or siPMVK for 48 h. Cell types were classified according to DEGs and known marker expression. The cells were divided into 13 clusters (Fig. 4a, Supplementary Fig. S5a). The relative cell proportions of Clusters 5, 7, 9, and 10 increased, whereas those of Clusters 0, 1, 2, 3, 4, 6, and 8 decreased in cells transfected with *miR-874* compared with control miRNA (Fig. 4a,b). In contrast, the proportions of Clusters 2, 5, 9 and 11 increased, whereas those of Clusters 0, 1, 3, 4, 6, 7, 8, 10, and 12 decreased in cells transfected with siPMVK compared with control siRNA (Fig. 4a,c). Gene Ontology (GO) terms associated with the cell cycle were enriched in approximately half of the 13 clusters (Supplementary Fig. S6). The increased proportions of Clusters 5 and 9 reflected common characteristics between cells transfected with *miR-874* and siPMVK. Cluster 5 contained genes involved in the cell cycle, and Cluster 9 contained genes related to the cellular response and p53 pathway (Supplementary Fig. S6). We examined cell cycle variations and visualized the cell cycle phases (Fig. 4d,e, Supplementary Fig. S5c). The proportions of S phase cells decreased among cells transfected with siPMVKs compared with control siRNA, whereas the proportions of G1 phase cells increased (Fig. 4d,e). No visible changes were observed among the cell cycle phases in cells transfected with *miR-874* compared with control miRNA, which was in contrast with the cell cycle analysis by flow cytometry (Figs. 3c, 4e). This difference may be due to the harvesting of cells just prior to cell death and the exclusion of dead cells from flow cytometry analysis. When we characterized clusters according to cell cycle phases, Clusters 0, 3 and 6 primarily consisted of S phase cells, whereas Clusters 2 and 9 were primarily G1 phase cells (Fig. 4f). These results are consistent with the finding that *miR-874* transfection induces S phase blockade and indicate that *PMVK* knockdown is associated with G1 arrest.

We investigated alterations in gene expression following *miR-874* or siPMVK transfection, especially p53 downstream genes and mevalonate pathway genes. First, we compared gene expression profiles among clusters, which showed variations in the enrichment of p53 target genes and mevalonate pathway genes (Supplementary Fig. S5d). To determine common components between bulk RNA-seq and scRNA-seq, we compared the DEGs, revealing 349 common DEGs between the two analyses (Fig. 4g), which were associated with biological process GO terms related to the cell cycle or cell death pathways (Fig. 4h). Furthermore, p53 and c-Myc were identified as upstream regulators based on transcription factor prediction analysis (Fig. 4i). Similar to the bulk RNA-seq results (Fig. 1), several p53 target genes were upregulated, and mevalonate pathway genes were downregulated in *miR-874*-transfected cells (Fig. 4j,k).

GO enrichment analysis and transcription factor enrichment analysis were applied to DEGs identified by scRNA-seq between cells transfected with control siRNA and siPMVK. In addition to the p53 pathway, GO enrichment analysis indicated enrichment of the cell cycle and apoptosis pathways in cells transfected with siPMVK compared with control siRNA (Supplementary Fig. S5e). Similar to the results for *miR-874*, p53 and c-Myc were identified as regulatory transcription factors for the identified DEGs (Fig. 4l). For data validation, the expression levels of *PMVK*, *SREBF2,* and *PPP1CA*, a previously reported *miR-874* target, were determined. *PMVK* expression was effectively suppressed by *miR-874* or siPMVK transfection (Fig. 4m, Supplementary Fig. S5b). *SREBF2* and *PPP1CA* expression was also downregulated by *miR-874* transfection (Fig. 4j,n, Supplementary Fig. S5b).

To examine common gene expression patterns between cells transfected with *miR-874* and *siPMVK,* we compared DEGs from the *miR-874* vs. control miRNA comparison with those identified in the siPMVK vs. control siRNA comparison (Supplementary Fig. S5f). Among the 89 identified common DEGs, GO enrichment analysis revealed the enrichment of cell cycle-associated pathways and p53 signalling pathways, including several p53 target genes upregulated by *PMVK* repression (Fig. 4k, Supplementary Fig. S5g). Consistent with our findings in Figs. 1, 2 and 3, *miR-874* expression altered the cell cycle and activated the p53 pathway. The gene expression profile for *miR-874* expression was similar to that for *PMVK* knockdown, suggesting that *miR-874* activates the p53 pathway at least partially through the suppression of the mevalonate pathway.

### Supplementation with isoprenoids partially rescues *miR-874*-induced apoptosis

**Figure 5:**
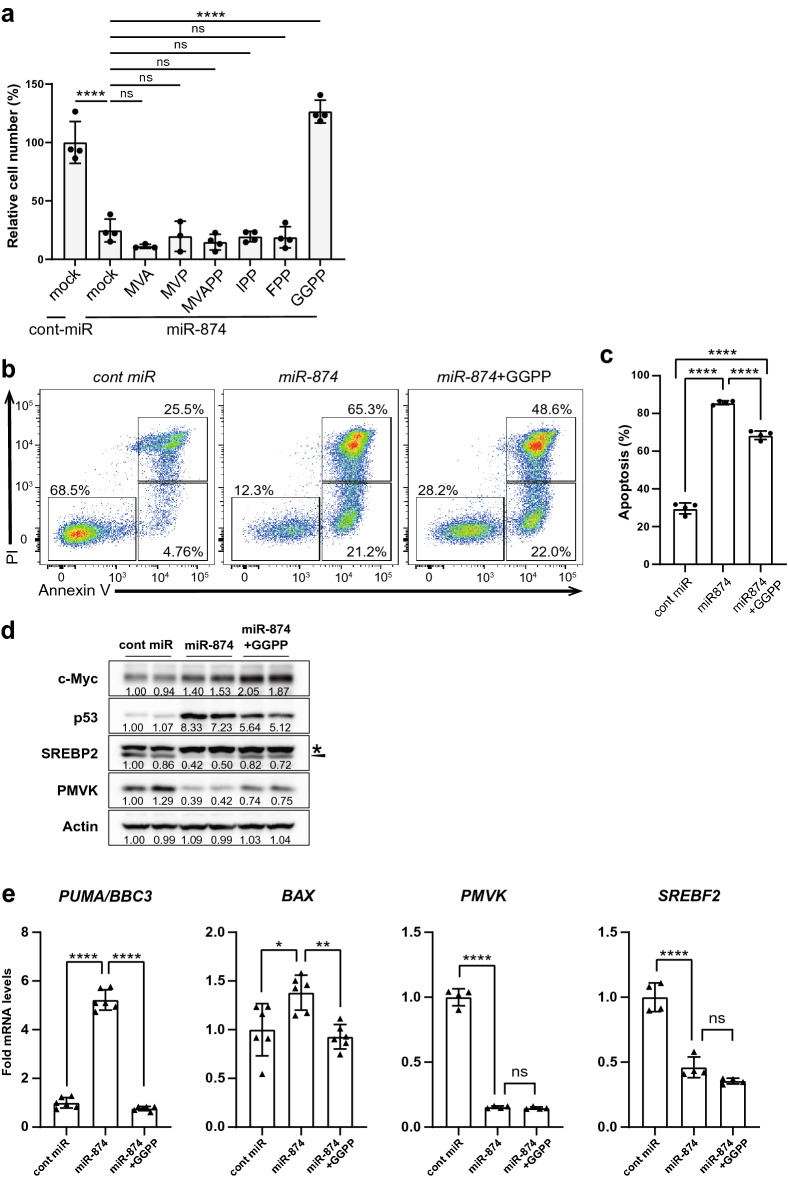
Supplementation with GGPP partially restores apoptosis induced by *miR-874.* (**a**) MCF-7 cells were transfected with control (cont) miRNA or *miR-874,* treated with mevalonate (MVA, 1 mM), mevalonate-5-phosphate (MVP, 0.5 mM), mevalonate-5-pyrophosphate (MVA-5PP, 0.5 mM), isopentenyl pyrophosphate (IPP, 15 μM), farnesyl pyrophosphate (FPP, 10 μM), or geranyl pyrophosphate (GGPP, 10 μM) for 72 h and subjected to the Alamar Blue assay. For mock samples, 70% methanol was added. Data are presented as the mean ± SD. ****p < 0.0001 (one-way ANOVA). (**b**) MCF-7 cells were transfected with cont miRNA or *miR-874*, treated with GGPP for 48 h, and subjected to annexin V-633/PI double staining with flow cytometry analysis. For the mock sample, 70% methanol was added. Representative images are shown. (**c**) The percentages of annexin V-633-negative/PI-negative cells (lower left, live cells), annexin V-633-positive/PI-negative cells (lower right, early apoptosis), and annexin V-633-positive/PI-positive cells (upper right, late apoptosis) were quantified. n = 4 per group. Data are presented as the mean ± SD. ****p < 0.0001 (one-way ANOVA). (**d**) MCF-7 cells were transfected with cont miRNA or *miR-874* and treated with GGPP for 40 h. Western blotting was performed to analyse the effects of *miR-874* transfection and GGPP treatment on c-Myc and p53 in MCF-7 cells. *indicates nonspecific bands, and triangles show bands for SREBP2. The numbers below the bands are the relative values of expression. (**e**) qRT-PCR analysis of *PMVK*, *SREBF2*, *BAX,* and *PUMA* in MCF-7 cells transfected with control or *miR-874* for 48 h. Data are presented as the mean ± SD (n = 6, *p < 0.05, **p < 0.01, ****p < 0.0001 by one-way ANOVA).

We investigated whether supplementation with mevalonate pathway metabolites could block *miR-874*-induced apoptosis. Cell proliferation was measured using Alamar Blue. Among the metabolites, only GGPP, which prenylates small GTPases and tethers them to plasma membranes, was able to restore cell growth following *miR-874* transfection (Fig. 5a). Unlike statins, which are inhibitors of HMGCR, *miR-874* acts on multiple genes in the cholesterol synthesis pathway, and treatment with mevalonic acid did not restore cell growth. To evaluate the effects of *miR-874* in MCF-7 cells, we investigated whether *miR-*874-induced apoptosis could be prevented through GGPP supplementation. Similar to previous results, *miR-874* significantly induced apoptosis in p53 WT MCF-7 cells, and GGPP treatment partially decreased the percentages of visibly detached cells and apoptotic cells, accompanied by an increase in the proportion of live cells (Fig. 5b,c). The p53 protein expression level, which increased in response to *miR-874* expression, decreased following GGPP supplementation (Fig. 5d). The expression levels of *PUMA* and *BAX*, which are downstream of p53 and increased in response to *miR-874* expression, decreased following GGPP supplementation (Fig. 5e). In contrast, the protein expression level of c-Myc increased in response to *miR-874* and was further increased by GGPP supplementation (Fig. 5d). These results indicate that the mevalonate pathway influences the apoptotic function of *miR-874*.

### Downregulation of PMVK inhibits MCF-7 breast cancer cell proliferation

**Figure 6:**
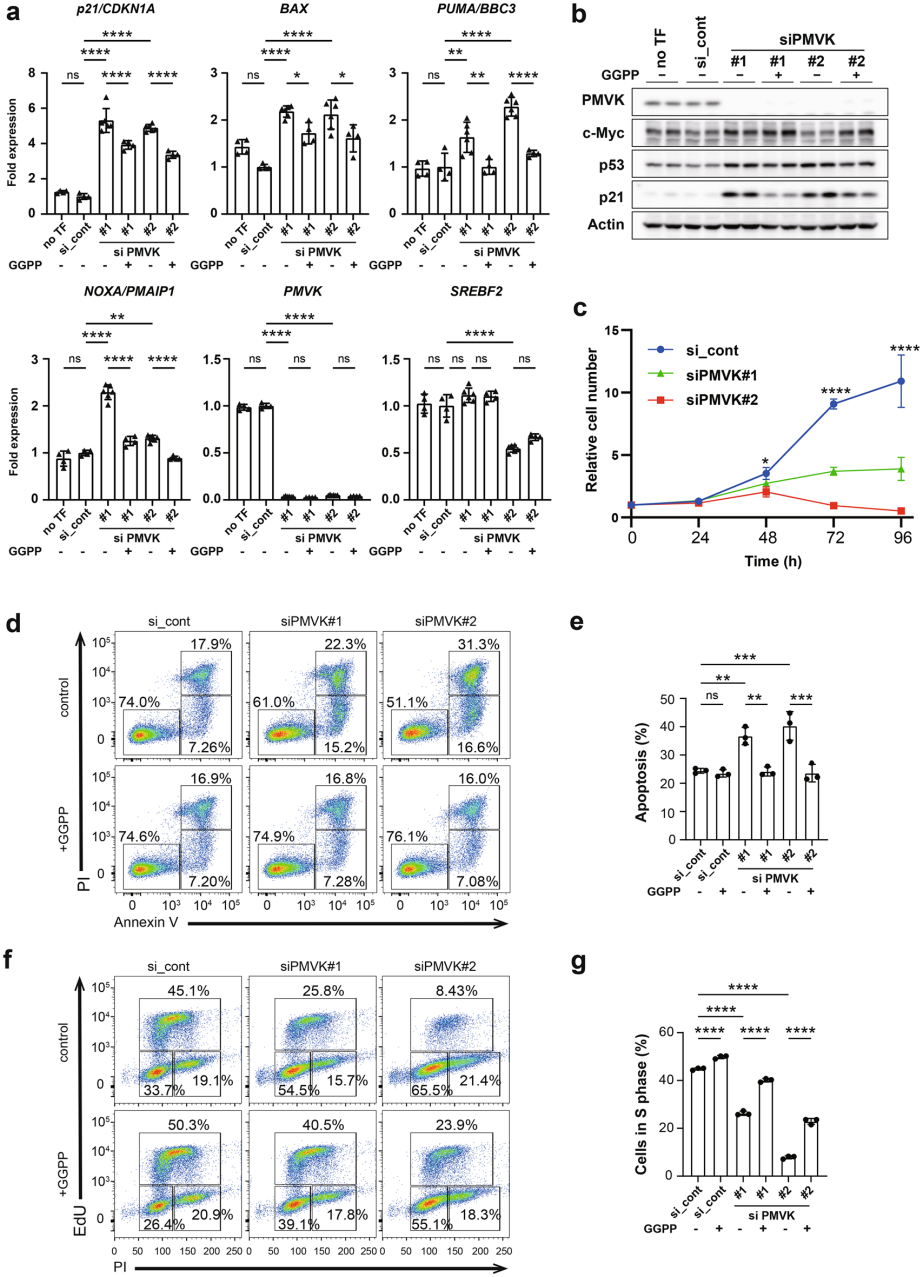
PMVK knockdown induces p53-mediated cell proliferation. (**a**) qRT‒PCR analysis of *PMVK*, *p21/CDKN1A, BAX*, *NOXA*, *PUMA,* and *SREBF2* in MCF-7 cells transfected with control or *PMVK* siRNAs and treated with or without geranylgeranyl pyrophosphate (GGPP) for 60 h (n = 4 or 6, mean ± SD, *p < 0.05, **p < 0.01, ****p < 0.0001 by one-way ANOVA). (**b**) Western blotting for PMVK, c-Myc, p53, p21/CDKN1A, and actin in MCF-7 cells transfected with control or *PMVK* siRNAs for 48 h. (**c**) p53 WT MCF-7 cells were transfected with control or *PMVK* siRNAs and subjected to a cell proliferation assay. Error bars represent the SD of the mean (n = 4, *p < 0.05, ****p < 0.0001 by one-way ANOVA). (**d**) MCF-7 cells were transfected with control or *PMVK* siRNAs for 64 h. The effect of *PMVK* knockdown on cell apoptosis was detected via annexin V-633/PI double staining of MCF-7 cells and flow cytometry assessment. Representative images are shown. (**e**) The percentages of annexin V-633-positive/PI-negative cells (lower right, early apoptosis) and annexin V-633-positive/PI-positive cells (upper right, late apoptosis) were quantified. (n = 3, **p < 0.01, ***p < 0.001 by one-way ANOVA). (**f**) MCF-7 cells were transfected with control or *PMVK* siRNA, treated with geranylgeranyl pyrophosphate (GGPP) for 48 h and subjected to an EdU assay with flow cytometry analysis. For the control sample, 70% methanol was added. Representative images are shown. (**g**) The proportion of cells in the S phase was quantitated and plotted (n = 3). Error bars represent the SDs of the means. ****p < 0.0001 (one-way ANOVA).

PMVK is a cytoplasmic enzyme that catalyses the conversion of mevalonate 5-phosphate into mevalonate 5-diphosphate in the mevalonate pathway^29^. To investigate the functional role of PMVK, we assessed the effects of silencing *PMVK* on apoptosis and the cell cycle in MCF-7 cells. The expression levels of PMVK mRNA and protein decreased following *PMVK* knockdown in MCF-7 cells (Fig. 6a,b). *p21/CDKN1A*, a cell cycle regulator downstream of p53, exhibited increased expression following *PMVK* knockdown. *BAX* and *PUMA*, apoptotic genes downstream of p53, were also affected by *PMVK* silencing (Fig. 6a,b). *PMVK* knockdown decreased the proliferation rate of p53 WT MCF-7 cells (Fig. 6c). Consistently, although *PMVK* knockdown did not increase the proportion of apoptotic MCF-7 cells after 48 h (data not shown), *PMVK* silencing induced apoptosis after 64 h (Fig. 6d,e). To test whether cell cycle arrest and the suppression of cell proliferation could be rescued by supplementation with mevalonate pathway metabolites, we treated *PMVK* knockdown cells with GGPP, which partially restored S phase progression (Fig. 6f,g). The GGPP treatment of *PMVK* knockdown MCF-7 cells also partially decreased *p21/CDKN1A*, *BAX,* and *NOXA* expression (Fig. 6a). These results suggest that *miR-874* suppresses the proliferation of breast cancer cells, at least in part, by inhibiting the mevalonate pathway via the downregulation of PMVK.

### High PMVK expression appears to be a risk factor in breast cancer patients

**Figure 7:**
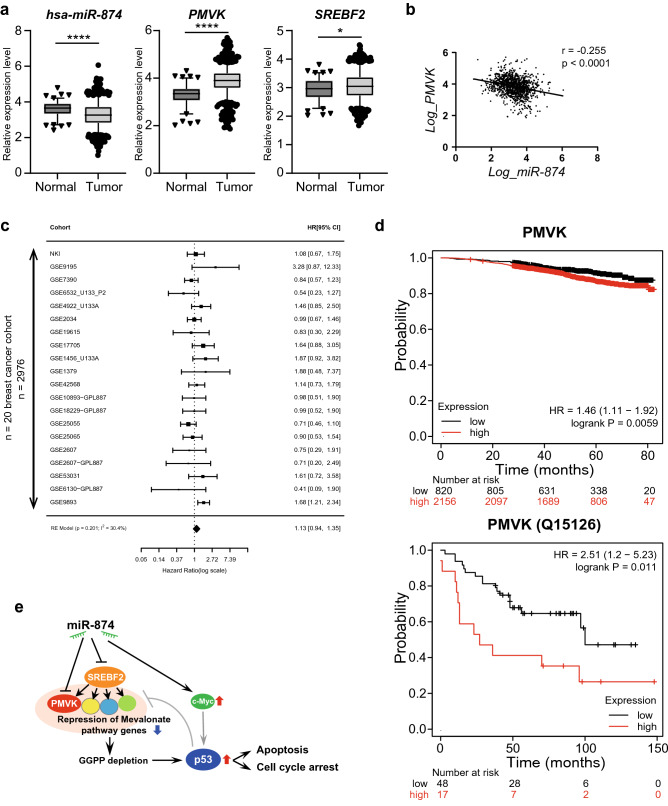
Expression of *miR-874*, *PMVK,* and *SREBF2* in The Cancer Genome Atlas (TCGA) database analysis and meta-analysis. (**a**) Expression of *miR-874* (left), *PMVK* (middle), and *SREBF-2* (right) in breast cancer tissues compared with normal tumour-adjacent tissues in the TCGA breast cancer dataset (normal samples [n = 104] versus tumour samples [n = 1093]). The data are presented as logarithmic values. The central line represents the median gene expression. Outliers above the 95th percentile and below the 5th percentile are displayed as dot plots. (**b**) Correlation analysis between the expression of *miR-874* and *PMVK* (Pearson’s r =  − 0.255, p < 0.0001). (**c**) Cohort studies for *PMVK* gene expression in a breast cancer meta-analysis were combined using random-effects models. The integrated hazard ratios (HRs) of relapse-free survival and its 95% confidence interval are shown. All cohorts were divided by the median gene expression ratio. Source data are provided in Supplementary Table S3. (**d**) Overall survival for the high and low PMVK mRNA expression groups (upper panel) and PMVK protein expression groups (lower panel) depicted using Kaplan–Meier plotter (https://kmplot.com/analysis). (**e**) A schematic view of a proposed mechanism through which miR-874 induces cancer cell death via suppression of the mevalonate pathway. miR-874 suppresses the mevalonate pathway by directly targeting PMVK and SREBF2, resulting in GGPP depletion, which activates the c-Myc and p53 signalling pathways and causes apoptosis and cell cycle arrest.

We confirmed the relationship between *miR-874* and *PMVK* expression and between *miR-874* and *SREBF2* expression in clinical specimens by analysing the TCGA breast cancer dataset. Both *PMVK* and *SREBF2* expression were elevated in tumour tissues (n = 1093) compared with adjacent normal tissues (n = 104), whereas *miR-874* expression was decreased in tumour tissues compared with corresponding normal tissues (Fig. 7a). Furthermore, *PMVK* expression was negatively correlated with *miR-874* expression (Fig. 7b), whereas the expression of *SREBF2* and other mevalonate pathway genes was not correlated with *miR-874* expression (Supplementary Fig. S7). We performed an integrated meta-analysis for *PMVK* using public datasets representing approximately 3000 patients with breast cancer (Fig. 7c). High *PMVK* expression tended to be associated with a poor prognosis (HR = 1.13) for relapse-free survival (not significant; Fig. 7c). Kaplan‒Meier survival analysis revealed that the overall survival of patients was worse in the high *PMVK* expression group than in the low PMVK expression group (p = 0.0059; Fig. 7d upper panel). The results of the analysis using protein expression showed a similar trend (p = 0.011, Fig. 7d lower panel), suggesting that *PMVK* expression might serve as a prognostic marker. On the other hand, for *SREBF2* mRNA, the overall survival analysis showed the opposite trend., i.e., the overall survival of patients was worse in the low *SREBF2 mRNA* expression group than in the high expression group (p = 5.4e−07; Supplementary Fig. S7b). A schematic model summarizing the study results is shown in Fig. 7e.

## Discussion

The mevalonate pathway is responsible for de novo cholesterol synthesis, in addition to the synthesis of many important nonsterol isoprenoid derivatives^30^. Elevated or dysregulated mevalonate pathway activity has been identified in various tumours, including breast cancer, and a number of studies have suggested that malignant cells are more dependent on the continuous availability of mevalonate pathway metabolites than their nonmalignant counterparts^31–33^. In this study, we focused on the functional role of *miR-874* in breast cancer and its association with the mevalonate pathway. *miR-874* induces apoptosis in MCF-7 cells in a p53-dependent manner, affecting the mevalonate pathway through the direct targeting of *PMVK* and *SREBF2*. RNA-seq analysis of *miR-874*-transfected MCF-7 cells identified the enrichment of p53 signaling pathways and the downregulation of several genes associated with the mevalonate pathway and *SREBF2*. scRNA-seq analysis revealed the effect of *miR-874* on the cell cycle. Some of the *miR-874* target genes previously reported are known to be involved in p53 activation. Regarding CDK9 function, suppression of CDK9 increases p53 stability. Inhibition of CDK9 not only reduces the MDM2-mediated ubiquitination and degradation of p53 but also increases p53 stability by suppressing the deacetylase activity of SIRT1^34^. E2F3 is a key repressor of the p19(Arf)-p53 pathway^35^. Regarding Stat3, Stat3 inhibits p53 gene transcription by binding to the p53 promoter^36^. Thus, *miR-874* may activate the p53 pathway via multiple target genes. Analysis of a microRNA expression panel of 51 breast cancer cell lines showed no significant difference in *miR-874* expression among the cell lines^37^, suggesting that the expression level of endogenous miR-874 in breast cancer cell lines does not differ between p53 WT and mutant. In p53 WT MCF-7 cells, *miR-874* overexpression resulted in cell death and cell cycle arrest. On the other hand, p53 mutant MDA-MB-231 did not induce cell death, cell cycle arrest or cell proliferation inhibition. *miR-874* overexpression suppressed cell proliferation in another p53-mutant triple-negative breast cancer cell line, MDA-MB-468^38^. The possibility that ER/PR + status and c-Myc have some influence could not be ruled out. Indeed, we tried to establish c-Myc single KO MCF-7-cell lines using CRISPR/Cas9. However, c-Myc KO cells were very slow to proliferate, and we could not maintain them. Apoptosis induction by *miR-874* overexpression completely abolished p53/c-Myc DKO (Fig. 3f).

Similar to *miR-874* overexpression, *PMVK* knockdown induced apoptotic cell death accompanied by p53 pathway activation, which could be partially restored by GGPP supplementation in MCF-7 cells. Although it is not known how *PMVK* knockdown causes apoptosis, PMVK has been associated with the development of porokeratosis, a group of keratinization disorders characterized by circular or annular skin lesions with a distinct hyperkeratotic rim termed the cornoid lamella^39^. In addition to *PMVK*, *MVD*, mevalonate kinase (*MVK*), *FDPS*, and *SLC17A9* are known as causative genes for porokeratosis^40–42^. A combination of cholesterol and statins that replace deficient end products, preventing the accumulation of potentially toxic metabolites, efficiently treats porokeratosis^43^. Premature apoptosis and incomplete keratinocyte differentiation were observed in the lesion tissues of *PMVK*-deficient patients^29^, and several studies have reported that p53 expression increases in the cornoid lamella of these lesions^44,45^, consistent with our results. Future studies may explore the mechanisms of apoptosis caused by inhibition of the mevalonate pathway. *PMVK* knockdown suppresses the downstream production of isoprenoids, such as farnesyl pyrophosphate (FPP) and GGPP, which are necessary for the synthesis of important biomolecules, such as cholesterol, dolichol and ubiquinone. FPP and GGPP isoprenylate small GTPases, such as Ras and Rho, which are involved in tumorigenesis, tethering these GTPases to the cell membrane for signal transduction^46^. GGPP and FPP inhibition induces GTPase dissociation from the cell membrane, suppressing Ras- and Rho-mediated signalling and inducing cancer cell death. Our results also showed that c-Myc expression increased following GGPP supplementation. Agabiti et al*.* reported that geranylgeranyl diphosphate synthase, which is an enzyme in the isoprenoid biosynthesis pathway that produces a pathway that produces GGPP, reduces the expression of Notch1 and affects the expression of its downstream target, c-Myc^47^.

The expression of PMVK in breast cancer tissue as reported in the TCGA database was higher than that in normal tissue adjacent to tumour tissue. Consistent with this finding, survival analysis in breast cancer showed that high *PMVK* expression was associated with poor prognosis. These results suggest that the suppression of breast cancer by the *miR-874*/*PMVK* axis may be relevant in many types of breast cancers, not just certain breast cancer cell lines. Although few previous reports have examined the association between PMVK and cancer, PMVK is reported to be differentially related to the multidrug response of ER-positive and ER-negative cells, with expression positively correlated with the drug response in ER-positive cells and negatively correlated with the drug response in ER-negative cells in breast cancer^48^, suggesting that *PMVK* might serve as a prognostic marker for breast cancer. In contrast, high *PMVK* expression is reported as a potential biomarker for favourable progression-free survival in ovarian cancer^49^. p53 status might also be associated with prognosis, and further clarification regarding which factors are involved in mediating antitumour effects remains necessary. *PMVK* knockdown induced apoptosis and cell cycle arrest, but the proportion of apoptotic cells was lower than that following *miR-874* transfection. A single miRNA can regulate dozens or even hundreds of target mRNAs^50,51^, and the downregulation of one target may have a minimal effect on apoptosis; however, our results indicate that *PMVK* downregulation has p53-dependent tumour-suppressive activity in breast cancer cells.

Unlike in the case of *PMVK, SREBF2* mRNA and *miR-874* expression did not show a correlation. Higher expression of *SREBF2* mRNA was associated with a better prognosis. When intracellular cholesterol levels are low, cleaving enzymes in the Golgi apparatus cut out the N-terminal region of SREBPs, and active SREBPs are transported to the nucleus, where they act as transcription factors to positively regulate the expression of genes related to cholesterol metabolism in the nucleus^52^. Therefore, *SREBF* mRNA levels do not necessarily reflect the protein expression or expression of genes related to cholesterol metabolism.

Several reports indicate that *miR-874* functions as a tumour suppressor and is downregulated in several cancers. The DNA methylation of the *miR-874* promoter region is upregulated in breast cancer tissues compared with normal tissues. Demethylation treatment with 5-Aza-2′-deoxycytidine (5-Aza-CdR) upregulated *miR-874* expression in breast cancer cell lines, suggesting that DNA methylation could be a mechanism of downregulation of *miR-874* expression^21^. There is also emerging evidence that long noncoding RNAs such as *lncRNA H19*, *lncRNA NEAT1*, *lncRNA miR210HG* and *lncRNA DCST1-AS1* act as sponges for *miR-874*, suppressing *miR-874* expression and contributing to cancer progression^53–56^. Further studies are needed to identify long noncoding RNAs that suppress the *miR-874*/*PMVK* axis. Our study revealed that *miR-874* activates the p53 signalling pathway by suppressing the mevalonate pathway via the depletion of GGPP, suggesting the therapeutic potential of *miR-874* for targeting the mevalonate pathway, especially in p53 WT breast cancers.

## Materials and methods

### Cell lines and cell culture

Two breast cancer cell lines, MCF-7 and MDA-MB-231, were obtained from the American Type Tissue Culture Collection (ATCC, Manassas, VA, USA). MCF-7 cells were cultured in Dulbecco’s modified Eagle medium (DMEM; Sigma-Aldrich, St Louis, MO, USA) supplemented with 10% (v/v) foetal bovine serum (FBS) and penicillin and streptomycin (Sigma-Aldrich). MDA-MB-231 cells were maintained in L-15 media (Thermo Fisher Scientific, Waltham, MA, USA) supplemented with 10% FBS and penicillin and streptomycin. p53 knockout (KO) and p53/c-Myc double-knockout (DKO) MCF-7 cells were generated by CRISPR‒Cas9 genome editing, as previously described^57^.

### miRNA transfection and small interfering RNA treatment

The following RNA species were used in this study: a pre-miRNA™ miRNA precursor (*hsa-miR-874*; premiR ID: PM12355) that mimics endogenous precursor miRNAs, two negative control (cont) miRNAs (P/N: AM17110 and AM17111; Thermo Fisher Scientific), small interfering RNAs (siRNAs; PMVK siRNA_1 and PMVK siRNA_2; Japan Bio Services, Saitama, Japan, and Thermo Fisher Scientific, respectively), and a negative control siRNA (Thermo Fisher Scientific) (Supplementary Table S2). RNA was reverse transfected into MCF-7 cells or MDA-MB-231 cells using RNAiMAX (Thermo Fisher Scientific) following the manufacturer’s protocol. Cells were harvested at the indicated time points after transfection and subjected to RNA sequencing (RNA-seq), apoptosis analysis, quantitative reverse-transcription polymerase chain reaction (qRT-PCR), dual-luciferase reporter assay, and western blot analysis.

### Chemical compounds and antibodies

Anti-actin antibody (#A2066), mevalonolactone (M4667), mevalonic acid 5-phosphate lithium salt hydrate (79849), mevalonic acid 5-pyrophosphate tetralithium salt (#94259), isopentenyl pyrophosphate triammonium salt (I0503), farnesyl pyrophosphate ammonium salt (F6892) and geranylgeranyl pyrophosphate ammonium salt (G6025) were purchased from Sigma-Aldrich. Anti-p21 (Ab1) antibody was purchased from Cell Signaling Technology (Danvers, MA, USA). Anti-PMVK (SC-390775) antibody was purchased from Santa Cruz Biotechnology (Santa Cruz, CA, USA). Anti-PMVK (15674-1-AP) antibody was purchased from Proteintech (Rosemont, IL, USA). Anti-c-Myc (ab32072), anti-SREBP2 (ab30682), and anti-p53 (DO1) antibodies were purchased from Abcam (Cambridge, UK).

### Apoptosis analysis

Harvested cells were stained with the Annexin V-633 Apoptosis Detection Kit (Nacalai Tesque, Kyoto, Japan) according to the manufacturer’s protocol. In brief, 1 × 10^5^ cells were incubated with 5 µl annexin V and 5 µl propidium iodide (PI) at room temperature for 15 min in the dark. The cells were analysed immediately with a flow cytometer [FACSCanto^TM^II, (BD Biosciences, Franklin Lakes, NJ, USA)].

### RNA-seq and data analysis

Total RNA was extracted from cells with the FastGene RNA Premium kit (Nippon Gene, Tokyo, Japan). RNA concentration and RNA integrity were measured using an Agilent 2100 bioanalyzer. Library construction and sequencing were performed as previously described^57^. RNA-seq libraries were prepared using the TruSeq Stranded mRNA Sample Prep Kit (Illumina, San Diego, CA, USA) following the manufacturer’s protocol. Deep sequencing was performed on the Illumina NextSeq 500 platform according to the manufacturer’s protocol.

Sequenced reads from RNA-seq experiments were aligned by using HISAT2, and Cufflinks was used for transcript assembly. Gene expression levels were expressed as fragments per kilobase of exon per million mapped sequence reads (FPKM). Differentially expressed genes (DEGs) were identified using the R/BioConductor software package (version 3.11)^58^. Heatmaps and dendrograms were generated by R software. DEGs related terms of Gene Ontology were analysed in Enrichr^59^, and the WikiPathway 2021 Human results were assessed. TRRUST Transcription Factors 2019 by Enrichr was used to identify the transcription factors that regulate both up- and downregulated genes.

### Single-cell RNA-seq library preparation and sequencing

Single-cell suspensions of MCF-7 cells were transfected with control miRNA or *miR-874* for 36 h or control siRNA or siPMVK for 48 h. Cells were washed twice with phosphate-buffered saline, trypsinized, and collected in DMEM. scRNA-seq libraries were prepared according to 10x Genomics specifications (Chromium Next GEM single-cell 3’ reagent kits and library construction kit, 10x Genomics, Pleasanton, CA, USA). Briefly, cell suspensions were loaded onto the 10x Genomics Chromium Controller to generate gel beads in emulsions (GEMs). After GEM generation, the samples were incubated at 53 °C for 45 min in a thermal cycler (Thermo Fisher Scientific) to generate polyA cDNA barcoded at the 5′ end by the addition of a template switch oligo linked to a cell barcode and unique molecular identifiers (UMIs). The GEMs were broken, and the single-strand cDNA was cleaned using DynaBeads My One Silane Beads (Thermo Fisher Scientific). cDNA was amplified (98 °C for 3 min; 11 cycles of 98 °C for 15 s and 63 °C for 20 s; 72 °C for 1 min), and cDNA quality was assessed using an Agilent TapeStation. cDNA was enzymatically fragmented, end-repaired, A-tailed, subjected to double-sided size selection with SPRIselect beads (Beckman Coulter, Indianapolis, Indiana, USA), and ligated to adaptors provided in the kit. A unique sample index for each library was introduced through 14 PCR amplification cycles using the indices provided in the kit (98 °C for 45 s; 14 cycles of 98 °C for 20 s, 54 °C for 30 s, and 72 °C for 20 s; 72 °C for 1 min; held at 4 °C). Indexed libraries were subjected to a second round of double-sided size selection, and libraries were quantified and quality assessed with an Agilent TapeStation. Libraries were submitted to GeneWiz (South Plainfield, NJ, USA), clustered using NovaSeq on a paired-end read flow cell, and sequenced on R1 (10x barcode and the UMIs), followed by 8 cycles of the I7 index (sample index) and 89 cycles based on R2 (transcript). 10x Genomics Cell Ranger Single Cell Software was used for sample demultiplexing, alignment with the human reference genome, GRCh38, filtering, UMI counting, single-cell 3′-end gene counting, and quality control using the manufacturer’s parameters^58^.

### scRNA-seq data analysis

The R package Seurat (v4.0) was used to cluster cells in a merged matrix. MCF-7 cells with > 10% mitochondrial gene expression were filtered out as low-quality cells. Individual gene counts for each cell were divided by the total gene counts for the cell and multiplied by a scale factor of 10,000; natural-log transformation was applied to all counts. The FindVariableFeatures function was used to select variable genes with default parameters. The ScaleData function was used to scale and centre the counts in the dataset. Principal component analysis was performed on variable genes, and 50 principal components were used for cell clustering (resolution = 5) and visualized as uniform manifold approximation and projection (UMAP) overlays, dot heat maps, and violin plots. A volcano plot was also constructed by R. Cluster markers were found using the FindAllMarkers function, and cell types were manually annotated based on cluster markers. We illustrated the cell cycle phase (G1, S, G2 M) using the CellCycleScoring function based on cell cycle-related genes^60^.

### Analysis of The Cancer Genome Atlas data

To investigate the clinical significance of miRNAs and genes in breast cancer, we used the RNA sequence database in TCGA (https://tcga-data.nci.nih.gov/tcga/). The gene expression and clinical data were obtained from the GDC Data Portal (https://portal.gdc.cancer.gov, the provisional data downloaded on 5th July 2020). *PMVK* and *miR-874* expression in an RNA-seq dataset was compared between normal (n = 104) samples and tumour (n = 1093) samples. For correlation analysis, Pearson’s correlation coefficients for *miR-874* gene expression versus *PMVK, SREBF2,* and other mevalonate pathway gene expression were calculated.

### Meta-analysis

We searched the public database PROGgeneV2, which compiles data associated with cohort studies from public repositories, including GEO, EBI Array Express, and TCGA. Studies through September 2021 with a study duration of > 5 years of relapse-free survival were selected for inclusion in our meta-analysis, resulting in 20 cohort studies entered into the meta-analysis.

Microarray or RNA sequencing data for *PMVK* in breast cancer are available online at PROGgeneV2 (http://www.progtools.net/gene/). Data were combined by means of random-effects models using the number of cases in each cohort as weights. The meta-analysis was performed using the R package *metafor*^61,62^. For Kaplan–Meier analysis, Kaplan–Meier plotter (https://kmplot.com/analysis) was used^63,64^.

### Statistical analysis

Quantitative data are presented as the mean ± standard deviation (SD). Comparisons between groups were performed by two-tailed Student’s t test or one-way analysis of variance (ANOVA) unless otherwise specified. The correlation between groups was determined by Pearson’s correlation analysis. JMP Pro 14 (https://www.jmp.com) and GraphPad Prism 9 software (https://www.graphpad.com) were used for statistical analyses. For Kaplan–Meier analysis, overall survival was assessed using the log-rank test for comparisons of the Kaplan–Meier event-free format using Kaplan–Meier plotter (https://kmplot.com/analysis)^63,64^. A p value of < 0.05 was considered significant.

## Supplementary Information

Supplementary Information 1.

Supplementary Information 2.

Supplementary Information 3.

Supplementary Information 4.

Supplementary Information 5.

Supplementary Information 6.

Supplementary Information 7.

Supplementary Information 8.

Supplementary Information 9.

Supplementary Information 10.

## Data Availability

The datasets generated and/or analyzed during the current study are available in the Gene Expression Omnibus (GEO) database, with accession number GSE214509. All data will be made available upon reasonable request. Please contact Tomoaki Tanaka (tomoaki@restaff.chiba-u.jp).
