# Supplementary material for: MicroRNA-874 targets phosphomevalonate kinase and inhibits cancer cell growth via the mevalonate pathway
Source: Sci Rep. 2022 Nov 2;12:18443. doi: 10.1038/s41598-022-23205-w (PMC9630378; doi:10.1038/s41598-022-23205-w)
Supplement: Supplementary file 2 — Supplementary Information 2. [file 41598_2022_23205_MOESM2_ESM.pdf]

Supplementary document

PMVK 3'UTR, 402 bp (wild type)

TCACTAGGTTCTAGGAGTGAGCTGGGGCCTGCTGAGGTGGGGGTGGGGCTGACTCTGCAAAATGGGGGTG
TCCCCCGATCCTGGCCGAGGTGAGGAACAGACAGGGGGGGTCTAGATTCTGAGGGGGTTGGTGGATATTG
GGCAAGGCAGGAAACCTCTGGAGACCTCATTTTCTCCATGGGGAAGACAGCCATGCTCTTCAGGAGGAGA
CTCCAAGGGCAAAGGAGGGTGTCTTGGCTGTGCTTGAAGGCGAAACCCTGCCATATCCCCAGTGCCAGTC
CCCTCAGCCTGTGGTGGCCTTGCATCCTGACTGGATGTTCTCAGCCCCTTGTTCTGGGCAAGAACCCAGA
GCTCCCCAGTGTGGATACTAATAAACCTCTTGGAGCACAGTTTAAACCTAGA

PMVK 3'UTR, 46 bp (Position 216-222 deletion)

TCACTAGGTTCTAGGAGTGAGCTGGGGCCTGCTGAGGTGGGGGTGGGGCTGACTCTGCAAAATGGGGGTG
TCCCCCGATCCTGGCCGAGGTGAGGAACAGACAGGGGGGGTCTAGATTCTGAGGGGGTTGGTGGATATTG
GGCAAGGCAGGAAACCTCTGGAGACCTCATTTTCTCCATGGGGAAGACAGCCATGCTCTTCAGGAGGAGA
CTCCA-------AGGAGGGTGTCTTGGCTGTGCTTGAAGGCGAAACCCTGCCATATCCCCAGTGCCAGTC
CCCTCAGCCTGTGGTGGCCTTGCATCCTGACTGGATGTTCTCAGCCCCTTGTTCTGGGCAAGAACCCAGA
GCTCCCCAGTGTGGATACTAATAAACCTCTTGGAGCACAGTTTAAACCTAGA

SREBF2 3'UTR, 412 bp (wild type)

CCACCAGGCTCAGCCCACCCCTCCACCTCTCTCTCGATTTCTCTCTCTCCCCCTCAGCATCTTCCCGCTG
AGAGTGGTGGGGAAGAGCCTTGTCTTCTTAGCTGTCACCTGCCGAGGCTTCTGGGCCACTCAGGCCAGTG
CACCCCTGGGCAGAGCCCCTTAAAGCTGCTGTCACTAGATGCCCATGGTCCAGGGCCTGGTGGGCGTGAG
AGGATAGGTGGCAGGGCAGAAACTGGGCAGCCCTGACTTGATAGCAGCAGGGGGAGCTCCCAAGCTGCCA
AGCCCCTGCCTCCAGCCTTCCTGAGTTTCTCTCTCCTGAACCCTACTCTCTCCTTTTTGCTTCCTCAGTT
TTTATCAGGCTTTCTCTGGGGGACAGCAGTCTCTGAGCACCAGGGAGCAGTTTAAACCTAGA

SREBF2 3'UTR, 405 bp (Position 222-228 deletion)

CCACCAGGCTCAGCCCACCCCTCCACCTCTCTCTCGATTTCTCTCTCTCCCCCTCAGCATCTTCCCGCTG
AGAGTGGTGGGGAAGAGCCTTGTCTTCTTAGCTGTCACCTGCCGAGGCTTCTGGGCCACTCAGGCCAGTG
CACCCCTGGGCAGAGCCCCTTAAAGCTGCTGTCACTAGATGCCCATGGTCCAGGGCCTGGTGGGCGTGAG
AGGATAGGTGG-------GAAACTGGGCAGCCCTGACTTGATAGCAGCAGGGGGAGCTCCCAAGCTGCCA
AGCCCCTGCCTCCAGCCTTCCTGAGTTTCTCTCTCCTGAACCCTACTCTCTCCTTTTTGCTTCCTCAGTT
TTTATCAGGCTTTCTCTGGGGGACAGCAGTCTCTGAGCACCAGGGAGCAGTTTAAACCTAGA
