# Supplementary material for: MicroRNA-874 targets phosphomevalonate kinase and inhibits cancer cell growth via the mevalonate pathway
Source: Sci Rep. 2022 Nov 2;12:18443. doi: 10.1038/s41598-022-23205-w (PMC9630378; doi:10.1038/s41598-022-23205-w)
Supplement: Supplementary file 3 — Supplementary Information 3. [file 41598_2022_23205_MOESM3_ESM.pptx]

## Slide 1
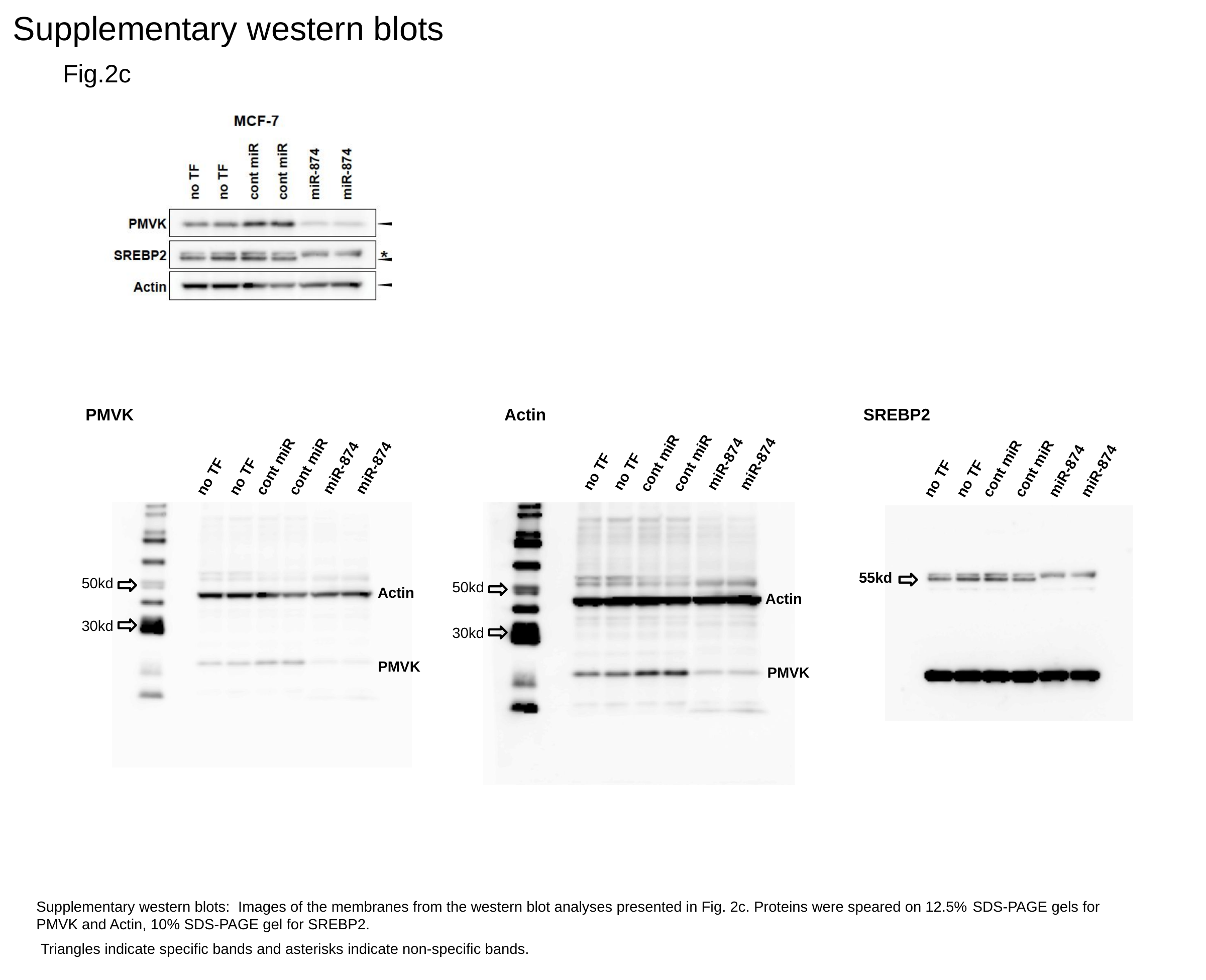

Supplementary western blots
Fig.2c
PMVK
Actin
SREBP2
cont miR
cont miR
miR-874
miR-874
cont miR
cont miR
miR-874
miR-874
cont miR
cont miR
miR-874
miR-874
no TF
no TF
no TF
no TF
no TF
no TF
55kd
50kd
50kd
Actin
Actin
30kd
30kd
PMVK
PMVK
cont miR
cont miR
miR-874
miR-874
no TF
no TF
Supplementary western blots: Images of the membranes from the western blot analyses presented in Fig. 2c. Proteins were speared on 12.5% SDS-PAGE gels for
PMVK and Actin, 10% SDS-PAGE gel for SREBP2.
Triangles indicate specific bands and asterisks indicate non-specific bands.

## Slide 2
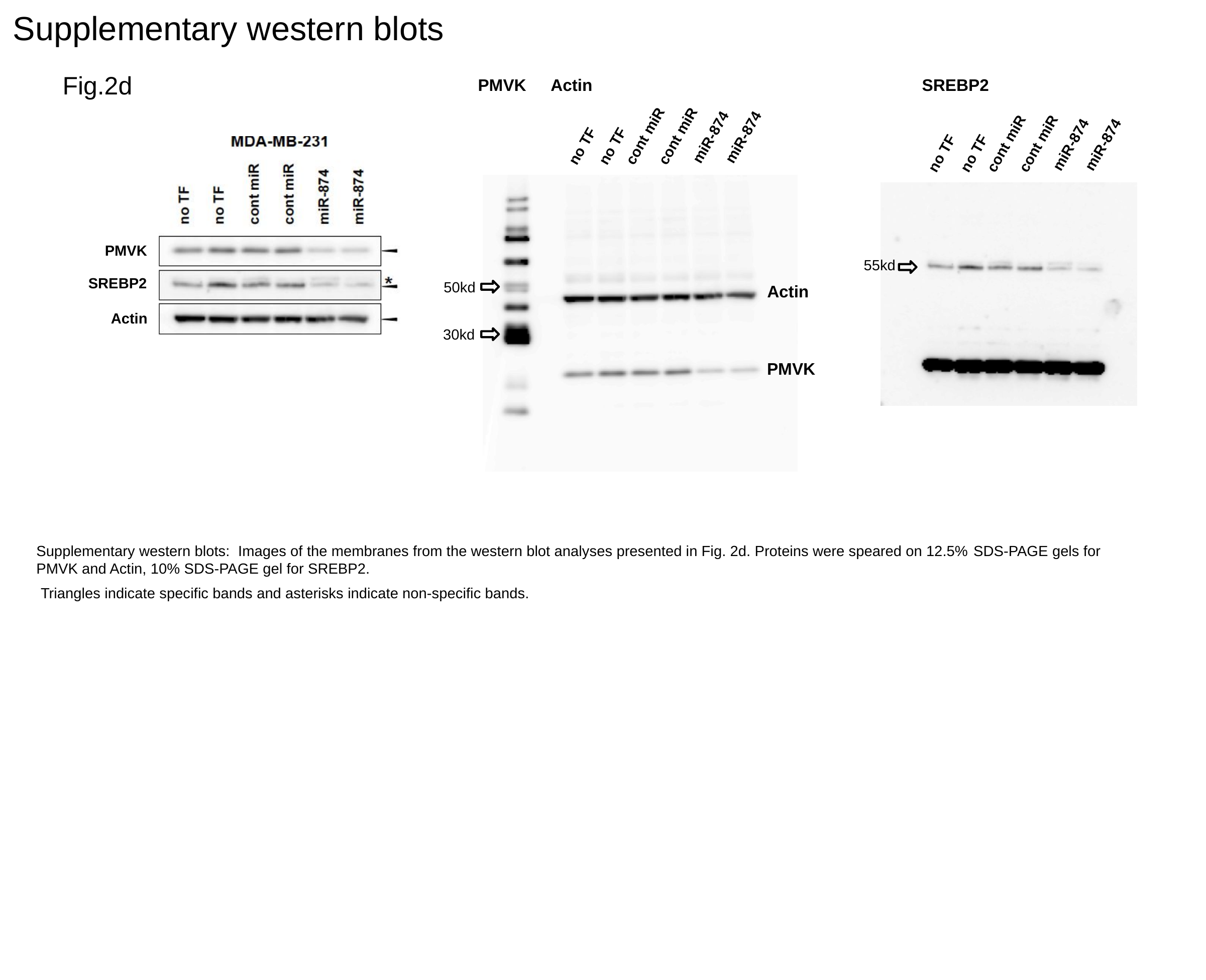

Supplementary western blots
Fig.2d
PMVK
Actin
SREBP2
cont miR
cont miR
miR-874
miR-874
no TF
no TF
50kd
Actin
30kd
PMVK
miR-874
miR-874
no TF
no TF
55kd
cont miR
cont miR
PMVK
SREBP2
Actin
Supplementary western blots: Images of the membranes from the western blot analyses presented in Fig. 2d. Proteins were speared on 12.5% SDS-PAGE gels for
PMVK and Actin, 10% SDS-PAGE gel for SREBP2.
Triangles indicate specific bands and asterisks indicate non-specific bands.
cont miR
cont miR
miR-874
miR-874
no TF
no TF

## Slide 3
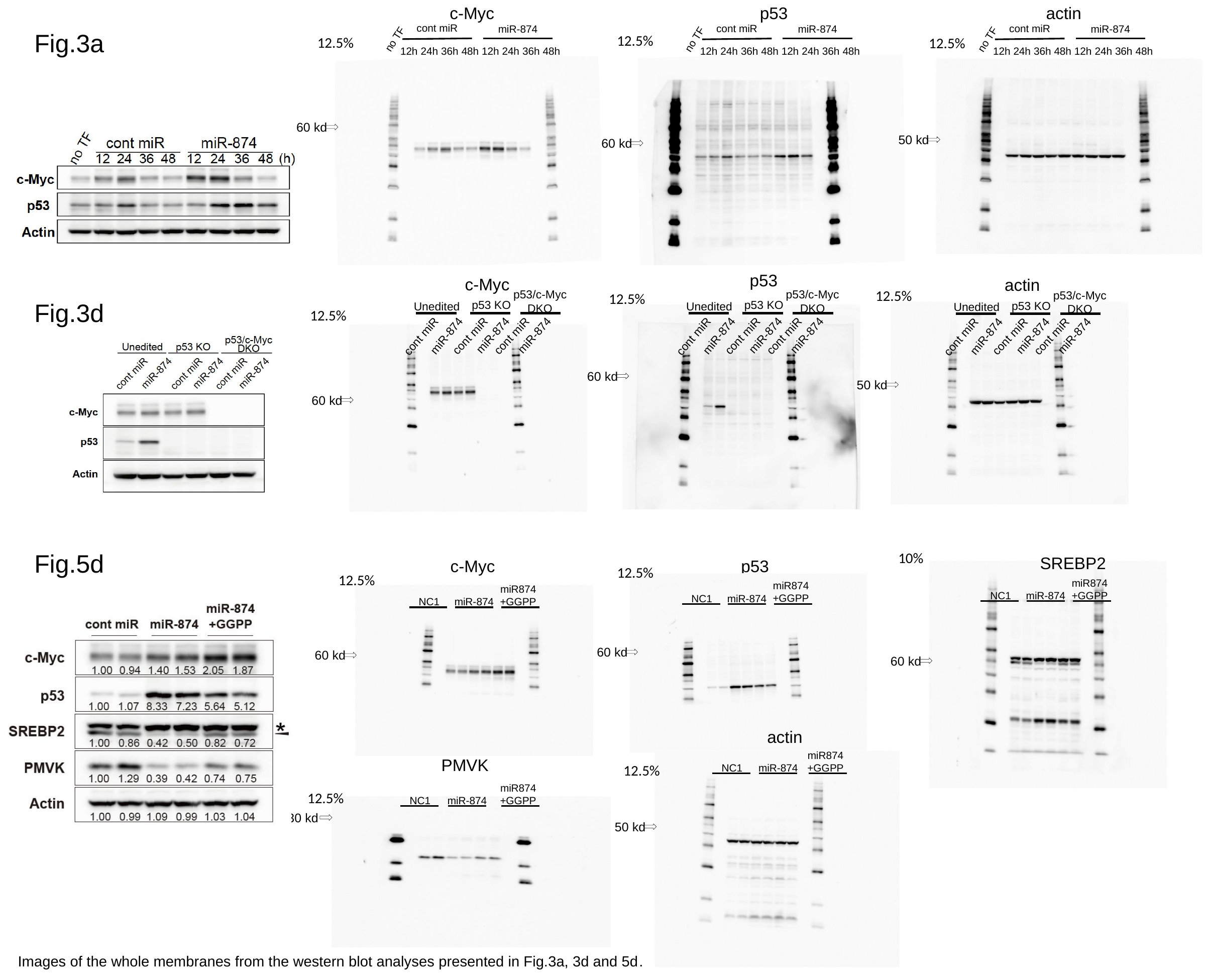

actin
c-Myc
p53
cont miR
cont miR
cont miR
miR-874
miR-874
miR-874
Fig.3a
12.5%
no TF
no TF
no TF
12.5%
12.5%
12h
24h
36h
48h
12h
24h
36h
48h
12h
24h
36h
48h
12h
24h
36h
48h
12h
24h
36h
48h
12h
24h
36h
48h
60 kd
50 kd
60 kd
p53
c-Myc
actin
12.5%
p53/c-Myc
DKO
p53/c-Myc
DKO
p53/c-Myc
DKO
12.5%
Fig.3d
p53 KO
p53 KO
p53 KO
Unedited
Unedited
Unedited
12.5%
miR-874
miR-874
miR-874
miR-874
miR-874
miR-874
cont miR
cont miR
cont miR
cont miR
miR-874
miR-874
cont miR
cont miR
miR-874
cont miR
cont miR
cont miR
60 kd
50 kd
60 kd
Fig.5d
10%
SREBP2
c-Myc
p53
12.5%
12.5%
miR874
+GGPP
miR874
+GGPP
miR874
+GGPP
NC1
miR-874
NC1
miR-874
NC1
miR-874
60 kd
60 kd
60 kd
actin
miR874
+GGPP
PMVK
12.5%
NC1
miR-874
miR874
+GGPP
12.5%
NC1
miR-874
30 kd
50 kd
Images of the whole membranes from the western blot analyses presented in Fig.3a, 3d and 5d.

## Slide 4
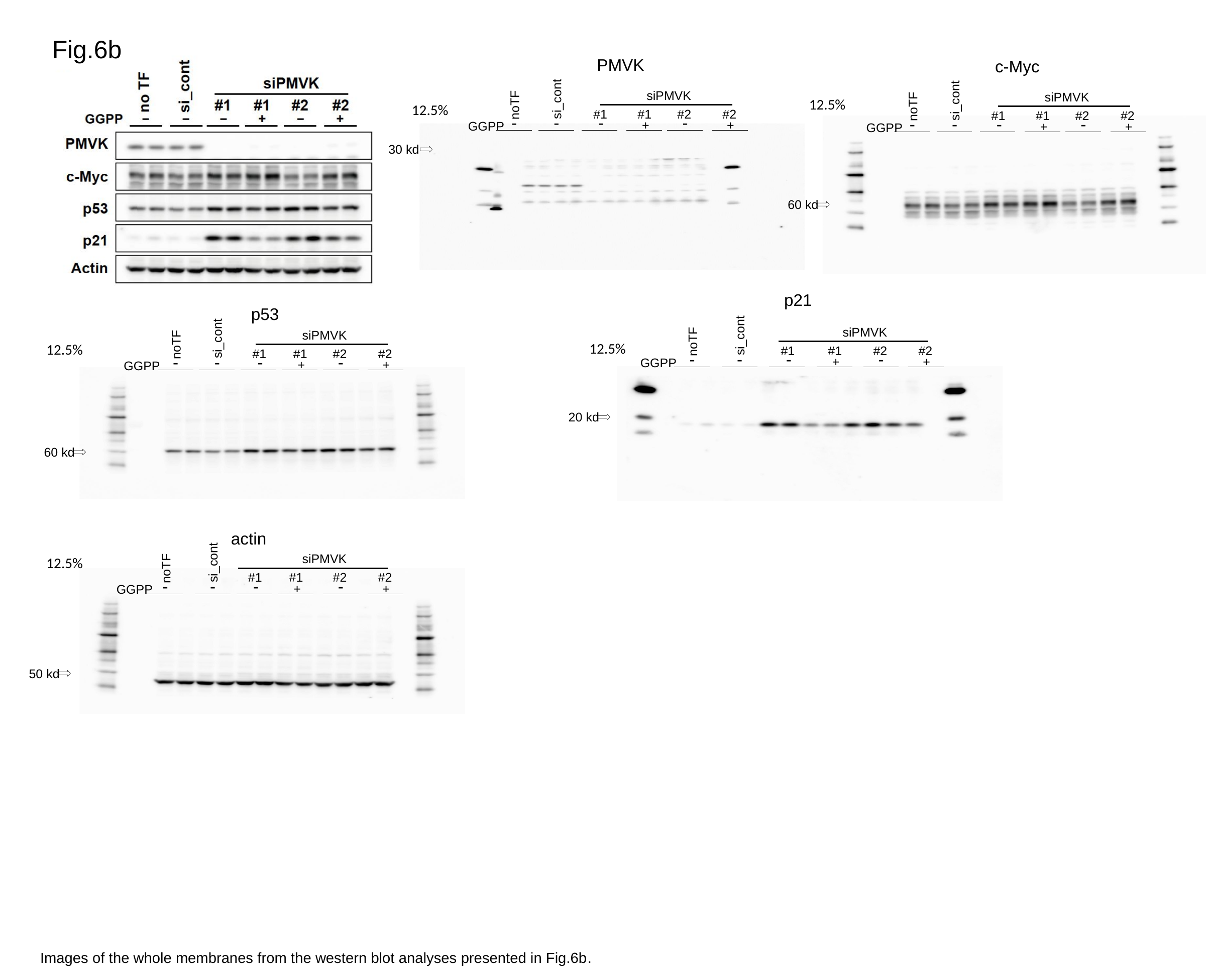

Fig.6b
PMVK
c-Myc
siPMVK
siPMVK
si_cont
si_cont
12.5%
noTF
noTF
12.5%
#1
#1
#2
#2
#1
#1
#2
#2
-
-
-
-
-
-
-
-
+
+
GGPP
+
+
GGPP
30 kd
60 kd
p21
p53
siPMVK
siPMVK
si_cont
si_cont
noTF
noTF
12.5%
12.5%
#1
#1
#2
#2
#1
#1
#2
#2
-
-
-
-
-
-
-
-
+
+
GGPP
+
+
GGPP
20 kd
60 kd
actin
siPMVK
12.5%
si_cont
noTF
#1
#1
#2
#2
-
-
-
-
+
+
GGPP
50 kd
Images of the whole membranes from the western blot analyses presented in Fig.6b.

## Slide 5
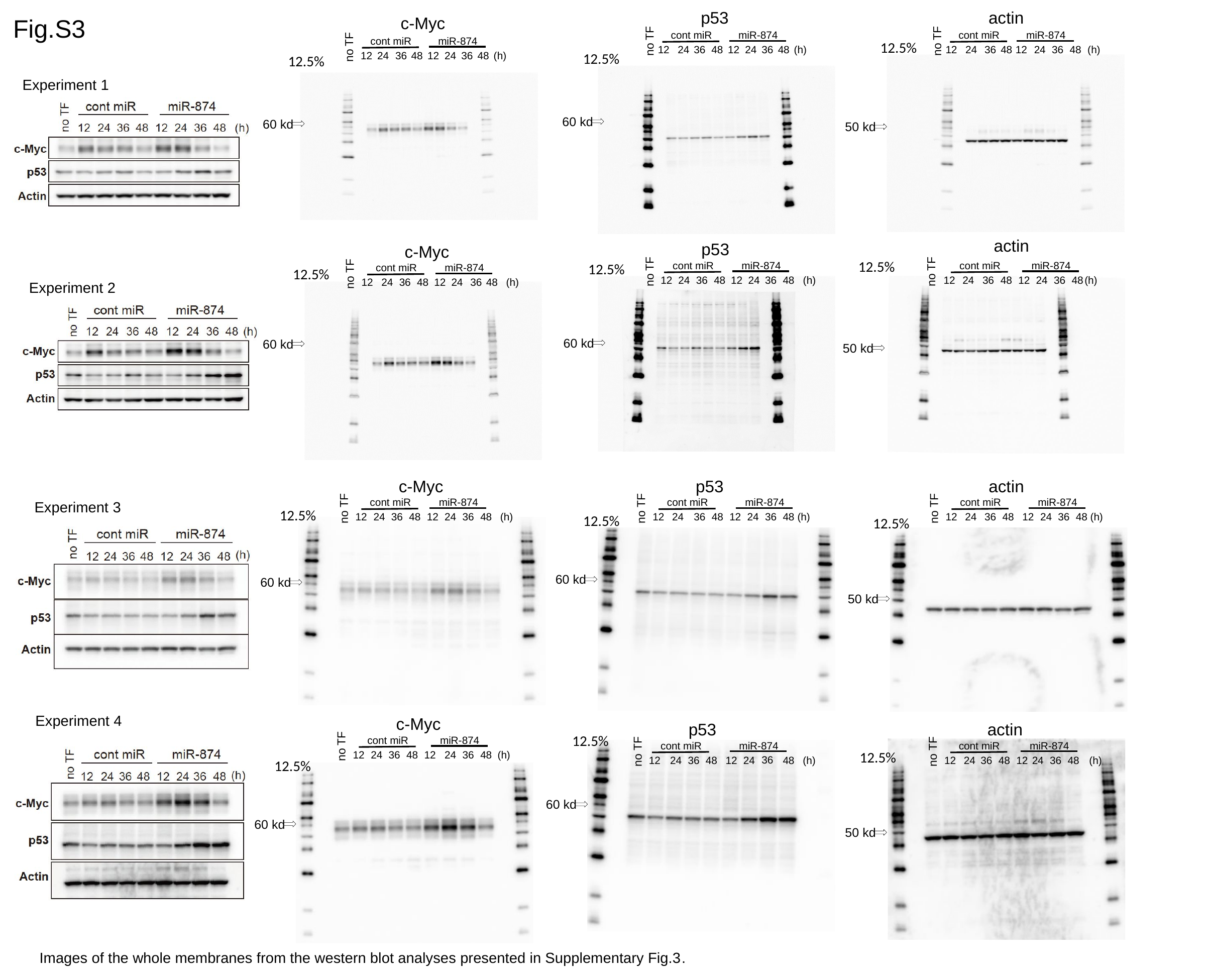

p53
actin
Fig.S3
c-Myc
cont miR
miR-874
cont miR
miR-874
no TF
no TF
cont miR
miR-874
no TF
12.5%
12
24
36
48
12
24
36
48
(h)
12
24
36
48
12
24
36
48
(h)
12
24
36
48
12
24
36
48
(h)
12.5%
12.5%
Experiment 1
60 kd
60 kd
50 kd
actin
p53
c-Myc
12.5%
cont miR
miR-874
cont miR
miR-874
12.5%
cont miR
miR-874
no TF
no TF
no TF
12.5%
12
24
36
48
12
24
36
48
(h)
12
24
36
48
12
24
36
48
(h)
12
24
36
48
12
24
36
48
(h)
Experiment 2
60 kd
60 kd
50 kd
c-Myc
p53
actin
cont miR
miR-874
cont miR
miR-874
cont miR
miR-874
Experiment 3
no TF
no TF
no TF
12.5%
12
24
36
48
12
24
36
48
(h)
12
24
36
48
12
24
36
48
(h)
12
24
36
48
12
24
36
48
(h)
12.5%
12.5%
60 kd
60 kd
50 kd
Experiment 4
c-Myc
p53
actin
12.5%
cont miR
miR-874
no TF
cont miR
miR-874
cont miR
miR-874
no TF
no TF
12
24
36
48
12
24
36
48
(h)
12.5%
36
12
24
36
48
12
24
48
(h)
12
24
36
48
12
24
36
48
(h)
12.5%
60 kd
60 kd
50 kd
Images of the whole membranes from the western blot analyses presented in Supplementary Fig.3.
