# Supplementary material for: MicroRNA-874 targets phosphomevalonate kinase and inhibits cancer cell growth via the mevalonate pathway
Source: Sci Rep. 2022 Nov 2;12:18443. doi: 10.1038/s41598-022-23205-w (PMC9630378; doi:10.1038/s41598-022-23205-w)
Supplement: Supplementary file 5 — Supplementary Information 5. [file 41598_2022_23205_MOESM5_ESM.pdf]

Supplementary table S2

List of siRNAs

Name	Target sequence
PMVK_1si	TTGGCTGTGCTTGAAGGCGAA
PMVK_2si	CCAGGCUUCUUUUGCAGGA
