# Supplementary material for: MicroRNA-874 targets phosphomevalonate kinase and inhibits cancer cell growth via the mevalonate pathway
Source: Sci Rep. 2022 Nov 2;12:18443. doi: 10.1038/s41598-022-23205-w (PMC9630378; doi:10.1038/s41598-022-23205-w)
Supplement: Supplementary file 6 — Supplementary Information 6. [file 41598_2022_23205_MOESM6_ESM.pdf]

Supplementary table S3

Source from Breast cancer										
outcome	relapse; Harzard ratio					data from PROGgeneV2				
PMVK	high		low							
cohort	Samples	# of events	Samples	# of events		HR	LCI (95%)	UCI (95%)	P VALUE	total sample
NKI	148	13	147	10		1.08	0.66	1.75	0.765	295
GSE9195	39	10	38	3		3.28	0.87	12.33	0.079	77
GSE7390	99	48	99	43		0.84	0.57	1.23	0.362	198
GSE6532_U133_P2	44	14	43	14		0.54	0.22	1.27	0.158	87
GSE4922_U133A	125	47	124	42		1.46	0.86	2.5	0.164	249
GSE2034	143	53	143	54		0.99	0.68	1.46	0.972	286
GSE19615	58	5	57	9		0.83	0.3	2.29	0.714	115
GSE17705	150	41	148	30		1.64	0.88	3.05	0.117	298
GSE1456_U133A	80	24	79	16		1.87	0.91	3.82	0.087	159
GSE1379	30	13	30	15		1.88	0.48	7.37	0.366	60
GSE42568	52	27	52	21		1.14	0.73	1.79	0.557	104
GSE10893-GPL887	26	9	25	10		0.98	0.51	1.9	0.961	51
GSE18229-GPL887	27	10	26	10		0.99	0.52	1.9	0.987	53
GSE25055	155	29	154	36		0.71	0.46	1.1	0.124	309
GSE25065	99	18	98	26		0.9	0.52	1.54	0.698	197
GSE2607	24	5	23	9		0.75	0.29	1.91	0.547	47
GSE2607-GPL887	19	6	19	6		0.71	0.2	2.49	0.594	38
GSE53031	83	22	83	19		1.61	0.73	3.58	0.239	166
GSE6130-GPL887	17	4	16	5		0.41	0.09	1.9	0.252	33
GSE9893	77	8	77	7		1.68	1.2	2.34	0.002	154
total	1495	406	1481	385						2976
