# Supplementary material for: MicroRNA-874 targets phosphomevalonate kinase and inhibits cancer cell growth via the mevalonate pathway
Source: Sci Rep. 2022 Nov 2;12:18443. doi: 10.1038/s41598-022-23205-w (PMC9630378; doi:10.1038/s41598-022-23205-w)
Supplement: Supplementary file 7 — Supplementary Information 7. [file 41598_2022_23205_MOESM7_ESM.pdf]

Supplementary table S4

List of Primers

Gene name	Sequence
β-ACTIN_Forward	CCTGGCACCCAGCACAAT
β-ACTIN_Reverse	GCCGATCCACACGGAGTACT
BAX_Forward	GATGCGTCCACCAAGAAGCT
BAX_Reverse	CGGCCCCAGTTGAAGTTG
FDPS_Forward	TGCAGAGTTCCTATCAGAC
FDPS_Reverse	AGGAAGGTAGAAGGAGTAG
HMGCR_Forward	GGAACCTCGGCCTAATGAAG
HMGCR_Reverse	CGAATAGATACACCACGCTCA
MVD_Forward	CGTGCTCATCCTTGTGGTG
MVD_Reverse	AAGCTGGGGAAGTCTCGCT
NOXA_Forward	GGCTGAGGTTCCCGGGCTCT
NOXA_Reverse	AGCGTTCTTGCGCGCCTTCT
p21/CDKN1A_Forward	GGCGGCAGACCAGCATGACAGATT
p21/CDKN1A_Reverse	GCAGGGGGCGGCCAGGGTAT
PMVK_Forward	GGAGGGCATCTCCCAGCCCA
PMVK_Reverse	GGCCCCATAGGCCTCCCGAA
PUMA_Forward	CCTGGAGGGTCCTGTACAATCT
PUMA_Reverse	GCACCTAATTGGGCTCCATCT
SREBF2_Forward	CCTGCTACTGTCGCTACGGA
SREBF2_Reverse	TTGCGGTATGCTGGGCGGAA
