# Supplementary material for: MicroRNA-874 targets phosphomevalonate kinase and inhibits cancer cell growth via the mevalonate pathway
Source: Sci Rep. 2022 Nov 2;12:18443. doi: 10.1038/s41598-022-23205-w (PMC9630378; doi:10.1038/s41598-022-23205-w)
Supplement: Supplementary file 8 — Supplementary Information 8. [file 41598_2022_23205_MOESM8_ESM.docx]

**Supplementary Figure S1. Expression of *miR-874* in p53 KO MCF-7 cells significantly downregulated mevalonate pathway metabolites, related to Figure 1.** (a) Principal component analysis showing clusters of WT, p53 KO and p53/c-Myc DKO MCF-7 cells, depending on the effect of *miR-874* transfection. Control (cont) miRNA was used for the control condition. n=3 per group. (b, c, d) Log ratio versus mean average (MA) plots show the results of differentially expressed gene (DEG) analysis comparing cells expressing cont miRNA and those expressing *miR-874* in (b) WT, (c) p53 KO and (d) p53/c-Myc DKO. (e, f) Top 10 pathways in common among the three cell types, p53 KO-specific and p53/c-Myc DKO-specific (e) up- and (f) downregulated DEGs induced by *miR-874* transfection were analysed by WikiPathway 2021. p values (Fisher’s exact test) are presented after the column. Genes that are commonly up- and downregulated in the three cell types are boxed in blue. Genes that are p53 KO-specific up- and downregulated are boxed in red. Genes that are p53/c-Myc DKO-specific up- and downregulated are boxed in orange. Pathways associated with the cell cycle are shown in bold.

**Supplementary Figure S2. *miR-874* downregulates the expression of multiple genes involved in the mevalonate pathway.** (a, b) qRT‒PCR assays were performed to measure the effects of *miR-874* on *HMGCR, MVD,* and *FDPS* mRNA in MCF-7 and MDA-MB-231 cells. Data are presented as the mean ± SD. n=6, *p<0.05, **p<0.01, ****p<0.0001 (one-way ANOVA).

**Supplementary Figure S3. *miR-874* sequentially activates c-Myc and p53. (Related to Figure 3).** Changes in the expression levels of c-Myc and p53 following *miR-874* transfection over time. MCF-7 cells were transfected with control (cont) miRNA or *miR-874*, harvested at the indicated time points, and subjected to western blotting. Western blotting images of four independent experiments are shown.

**Supplementary Figure S4. *miR-874* does not induce apoptosis but suppresses the proliferation of MDA-MB-231 cells.** (a) MDA-MB-231 cells were transfected with control (cont) miRNA or *miR-874*. After 48 h of transfection, the proportions of apoptotic cells were measured by flow cytometric analysis of cells stained with annexin V-633 and PI (n=3 per group). Representative images are shown. (b) The percentages of annexin V-633–positive/PI-negative cells (lower right, early apoptosis) and annexin V-633–positive/PI-positive cells (upper right, late apoptosis) were quantified. Annexin V-positive cells: cont miRNA; 9.0% vs. *miR-874*; 9.9%. (c) Cell proliferation curve after transfection of *miR-874* at the indicated time points in MDA-MB-231 cells. The error bars represent the SD of the mean (n=5, ** p<0.01, Student’s t test). (d) MDA-MB-231 cells were transfected with control (cont) miRNA or *miR-874* and subjected to an EdU assay after 48 h using flow cytometry analysis. Representative images are shown. (e) The proportions of cells in the S phase, G0/G1 phase, and G2/M phase were quantitated and plotted (n=3). Error bars represent the SDs of the means (S phase: cont miRNA; 31.4% vs. *miR-874*; 21.3%, p<0.01, Student’s t test).

**Supplementary Figure S5. scRNA-seq from siPMVK revealed that the cellular targets of *miR-874* coincide with siPMVK.** (a) scRNA-seq data (n=46,130) across all four groups of treated MCF-7 cells are shown as nonlinear representations of the top 50 principal components; cells are shown in different colours according to UMAP-based clusters. (b) UMAP plots of MCF-7 cells show the expression of *miR-874* target genes (*PMVK*, *SREBF2*, and *PPP1CA*). (c) Cells are shown in different colours according to UMAP-based clusters according to cell phase. (d) The top 120 differentially expressed genes (DEGs) defined by the FindAllMarkers function from each cluster are listed in the heatmap. Representative p53 target genes are shown in red, whereas mevalonate pathways are shown in blue in the list. (e) The 241 DEGs identified by scRNA-seq between control siRNA and siPMVK were analysed by Gene Ontology. The table shows multiple pathways enriched in the 241 DEGs, as analysed by Metascape. (f) Venn diagram showing 89 common DEGs from the 437 genes identified between control mRNA and *miR-874* and 241 genes identified between control siRNA and siPMVK according to scRNA-seq analysis. (g) The table shows multiple pathways enriched in the 89 common DEGs, as analysed by Metascape.

**Supplementary Figure S6. The results of Gene Ontology analysis from each cluster in the scRNA-seq analysis.** Differentially expressed genes (DEGs) in each cluster were defined by the FindAllMarkers function. The DEGs for each cluster were analysed by Metascape, and multiple pathways are listed. Clusters that share common characteristics between the cells transfected with *miR-874* and siPMVK are marked with red frames.

**Supplementary Figure S7. Correlations between *miR-874* and mevalonate pathway gene expression in the TCGA breast database.** (a) Correlation analysis between the expression of *miR-874* and mevalonate pathway genes by Pearson’s correlation coefficient (*ACAT2*, *HMGCS1*, *HMGCR*, *MVK*, *MVD,* *FDPS* and *SREBF2*). (b) Overall survival for the high and low *SREBF2 mRNA* expression groups depicted using Kaplan–Meier plotter (<https://kmplot.com/analysis>).
