# Supplementary material for: MicroRNA-874 targets phosphomevalonate kinase and inhibits cancer cell growth via the mevalonate pathway
Source: Sci Rep. 2022 Nov 2;12:18443. doi: 10.1038/s41598-022-23205-w (PMC9630378; doi:10.1038/s41598-022-23205-w)
Supplement: Supplementary file 9 — Supplementary Information 9. [file 41598_2022_23205_MOESM9_ESM.docx]

**Supplementary Materials and Methods**

**5-Ethynyl-2’-deoxyuridine analysis**

The 5-ethynyl-2’-deoxyuridine (EdU) assay (Click-iT Plus EdU Flow Cytometry Assay Kits; C10632, Thermo Fisher Scientific) was performed according to the manufacturer’s protocol. In brief, cells were incubated with 10 µM EdU for 1 hour. Cells were harvested, fixed with 4% paraformaldehyde, stained with Alexa Fluor 488 for the detection of EdU and 4′,6-diamidino-2-phenylindole (DAPI) for cell cycle assessment, and analysed by flow cytometry.

**Alamar Blue analysis**

MCF-7 cells were transfected with control miRNA or *miR-874*, treated with mevalonate pathway metabolites for 72 h, and subjected to the Alamar Blue assay. Alamar Blue (AlamarBlue™ HS Cell Viability Reagent, A50100, Thermo Fisher Scientific) was mixed with MCF-7 media at a 1:10 ratio, added to cells, and incubated at 37 °C for 6 h. Absorbance readings were obtained at 570 and 600 nm.

**qRT‒PCR**

After the cells were harvested, total RNA was isolated with TRIzol (Thermo Fisher Scientific) or the RNeasy Mini Kit (Qiagen, Valencia, CA, USA) and subsequently reverse transcribed into cDNA with the ReverTra Ace® qPCR RT Kit (TOYOBO, Osaka, Japan). *miR-874* expression levels were detected with a TaqMan miRNA assay (Assay ID: 002268; Thermo Fisher Scientific) and normalized according to *RNU48* expression (Assay ID: 001006; Thermo Fisher Scientific); the expression levels of other factors were assessed by the 2^−ΔΔCt^ method ^1^. qRT‒PCR was performed on a StepOnePlus^TM^ System (Thermo Fisher Scientific) with the default fast mode setting (stage 1; 95.0 °C for 20 s, stage 2; 40 cycles at 95.0 °C for 1 s and 60.0 °C for 20 s). Primers are shown in Supplementary Table S4.

**Western blot analysis**

Cells were lysed in lysis buffer (25 mM Tris-HCl, pH 7.4; 150 mM NaCl; 1% NP-40; 1 mM EDTA; 5% glycerol) supplemented with protease inhibitor cocktail and phosphatase inhibitor cocktail. The lysates were separated by sodium dodecyl sulfate–polyacrylamide gel electrophoresis and transferred to polyvinylidene fluoride membranes. The membranes were sequentially blocked with blocking reagent (10% skimmed milk and 1% BSA), incubated with the appropriate primary antibodies according to the manufacturer's protocol, and incubated with horseradish peroxidase-conjugated secondary antibodies (Agilent Technologies, Santa Clara, CA, USA and GE Healthcare, Boston, MA, USA). Immunoreactive bands were visualized by enhanced chemiluminescence (Thermo Fisher Scientific), and images were acquired with a LAS-4000 Mini imaging system (Cytiva, Grens, Switzerland) or Fusion FX (Vilber Lourmat, Collégien, France). Protein band intensities were quantitated using Evolution Caput software.

**Plasmid construction and dual-luciferase reporter assay**

Predicted target genes and their target miRNA binding site seed regions were determined using TargetScan (<http://www.targetscan.org/>) and STarMir (<https://sfold.wadsworth.org/>). The wild-type (WT) sequences for the *PMVK* 3’-untranslated region (UTR) and the *SREBF2* 3’-UTR were inserted between the *Xho*I–*Not*I restriction sites in the *hRluc* 3’-UTR in the psiCHECK-2 vector (C8021; Promega, Madison, WI, USA). Plasmid vectors encoding the *PMVK* 3’-UTR with deleted *miR-874* target sites (position 216–222; NM_006556) or the *SREBF2* 3’-UTR with deleted *miR-874* target sites (position 222–228; NM_004599) were generated using KOD-FX (KFX-101, TOYOBO) and DNA assembly (M5520, New England BioLabs, Ipswich, MA, USA). Oligonucleotide sequences are described in the Supplementary document. A dual-luciferase reporter assay was performed as described previously ^2,3^, with some modifications. p53/c-Myc DKO MCF-7 cells were transfected with 40 ng vector using 0.5 µl Lipofectamine 2000 (Thermo Fisher Scientific) in 50 µl Opti-MEM 1 (Thermo Fisher Scientific) and 10 nM *miR-874* using 0.5 µl RNAiMAX (Thermo Fisher Scientific) in 50 µl Opti-MEM 1 (Thermo Fisher Scientific). After 44–45 h of transfection, the firefly and Renilla luciferase activities of cell lysates were determined using a dual-luciferase assay system (E1910; Promega). Data were normalized by calculating the Renilla luciferase activity/firefly luciferase activity ratio.

**References**

1 Ochiiwa, H. *et al.* TAS4464, a NEDD8-activating enzyme inhibitor, activates both intrinsic and extrinsic apoptotic pathways via c-Myc-mediated regulation in acute myeloid leukemia. *Oncogene* **40**, 1217-1230, doi:10.1038/s41388-020-01586-4 (2021).

2 Nohata, N. *et al.* Tumour suppressive microRNA-874 regulates novel cancer networks in maxillary sinus squamous cell carcinoma. *Br J Cancer* **105**, 833-841, doi:10.1038/bjc.2011.311 (2011).

3 Nohata, N. *et al.* Tumour-suppressive microRNA-874 contributes to cell proliferation through targeting of histone deacetylase 1 in head and neck squamous cell carcinoma. *Br J Cancer* **108**, 1648-1658, doi:10.1038/bjc.2013.122 (2013).
