# Supplementary material for: MicroRNA-874 targets phosphomevalonate kinase and inhibits cancer cell growth via the mevalonate pathway
Source: Sci Rep. 2022 Nov 2;12:18443. doi: 10.1038/s41598-022-23205-w (PMC9630378; doi:10.1038/s41598-022-23205-w)
Supplement: Supplementary file 10 — Supplementary Information 10. [file 41598_2022_23205_MOESM10_ESM.pdf]

Figure S1

a **b** **c** **d**

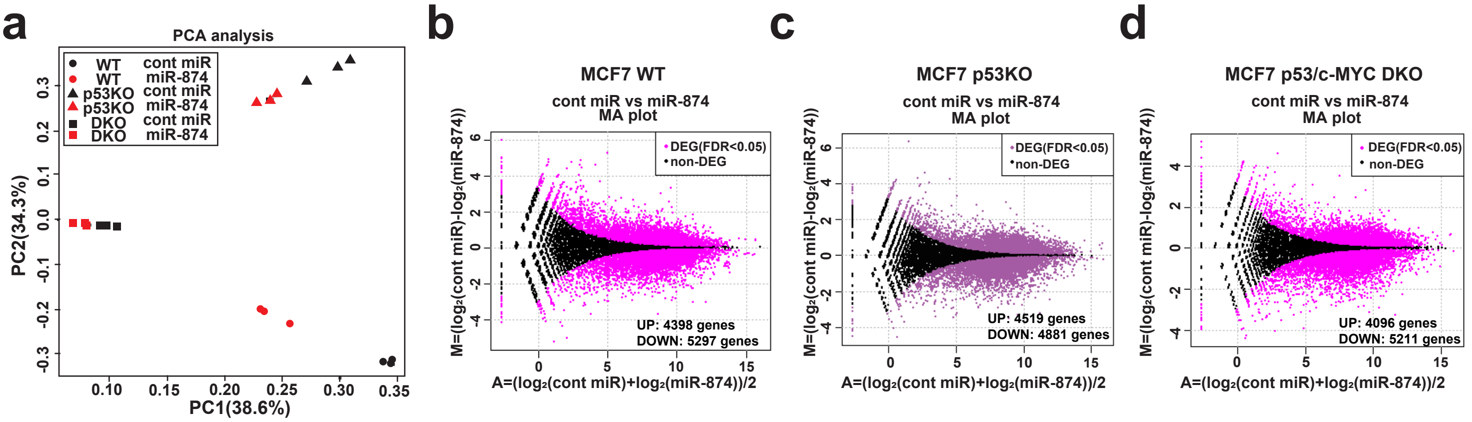

e

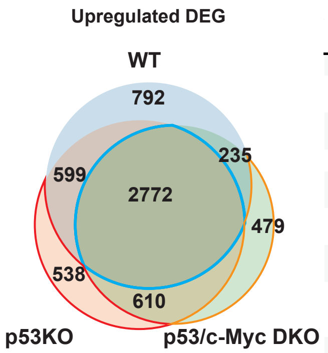

WikiPathway 2021 Human

common upregulated
2772 genes

Index	Name	P-value
1	mRNA Processing WP411	5.07E-23
2	Eukaryotic Transcription Initiation WP405	4.25E-08
3	DNA Repair Pathways Full Network WP4946	4.54E-08
4	DNA Replication WP466	1.52E-07
5	Pyrimidine metabolism WP4022	0.000001816
6	Nucleotide Excision Repair WP4753	0.000002011
7	Cytoplasmic Ribosomal Proteins WP477	0.000005175
8	DNA IR-double strand breaks and cellular response via ATM WP3959	0.000006376
9	**Cell cycle WP179**	0.000009704
10	DNA IR-damage and cellular response via ATR WP4016	0.00001648

p53KO specific upregulated
538 genes

Index	Name	P-value
1	LDLRAD4 and what we know about it WP4904	0.0006249
2	Insulin signaling in adipocytes (diabetic condition) WP3635	0.0009799
3	Insulin signaling in adipocytes (normal condition) WP3634	0.0009799
4	Type II diabetes mellitus WP1584	0.002576
5	TGF-beta Receptor Signaling WP560	0.003186
6	TGF-beta receptor signaling in skeletal dysplasias WP4816	0.004564
7	Proteasome Degradation WP183	0.006336
8	Mesodermal commitment pathway WP2857	0.006502
9	Extracellular vesicle-mediated signaling in recipient cells WP2870	0.008148
10	Nonalcoholic fatty liver disease WP4396	0.009303

DKO specific upregulated
479 genes

Index	Name	P-value
1	Hypothesized Pathways in Pathogenesis of Cardiovascular Disease WP3668	0.00002199
2	VEGFA-VEGFR2 Signaling Pathway WP3888	0.0001296
3	Association Between Physico-Chemical Features and Toxicity Associated Pathways WP3680	0.0001735
4	DNA damage response WP707	0.0002142
5	miRNA regulation of DNA damage response WP1530	0.0002896
6	Focal Adhesion WP306	0.0003124
7	**Cell cycle WP179**	0.0006263
8	BDNF signaling pathway WP2380	0.000711
9	Regulation of Actin Cytoskeleton WP51	0.0009964
10	**G1 to S cell cycle control WP45**	0.004243

f

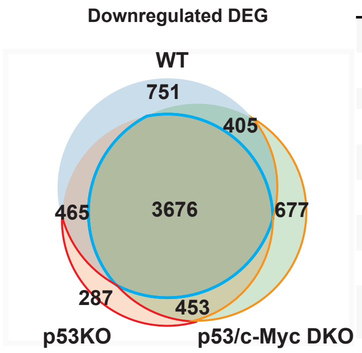

WikiPathway 2021 Human

common downregulated
3676 genes

Index	Name	P-value
1	EGF/EGFR signaling pathway WP437	2.17E-13
2	Gastrin signaling pathway WP4659	2.97E-10
3	Insulin Signaling WP481	1.13E-09
4	VEGFA-VEGFR2 Signaling Pathway WP3888	3.29E-09
5	TSH signaling pathway WP2032	5.57E-09
6	Regulation of Microtubule Cytoskeleton WP2038	4.62E-08
7	Angiopoietin Like Protein 8 Regulatory Pathway WP3915	2.85E-07
8	Pancreatic adenocarcinoma pathway WP4263	9.90E-07
9	Translation inhibitors in chronically activated PDGFRA cells WP4566	0.000001135
10	Ciliopathies WP4803	0.000001976

p53KO specific downregulated
287 genes

Index	Name	P-value
1	Mechanoregulation and pathology of YAP/TAZ via Hippo and non-Hippo mechanisms WP4534	0.0005505
2	Inflammatory Response Pathway WP453	0.000848
3	Integrated Cancer Pathway WP1971	0.00359
4	Senescence and Autophagy in Cancer WP615	0.004075
5	Hippo-Yap signaling pathway WP4537	0.004187
6	DNA IR-damage and cellular response via ATR WP4016	0.005884
7	Nanoparticle-mediated activation of receptor signaling WP2643	0.007347
8	**Cell cycle WP179**	0.007748
9	DNA IR-double strand breaks and cellular response via ATM WP3959	0.007979
10	Mammary gland development pathway - Involution (Stage 4 of 4) WP2815	0.008558

DKO specific downregulated
677 genes

Index	Name	P-value
1	Type I interferon induction and signaling during SARS-CoV-2 infection WP4868	0.00006385
2	IL-10 Anti-inflammatory Signaling Pathway WP4495	0.0005184
3	Host-pathogen interaction of human coronaviruses - interferon induction WP4880	0.0007462
4	Immune response to tuberculos WP4197	0.0008872
5	Genotoxicity pathway WP4286	0.001239
6	Type II interferon signaling (IFNG) WP619	0.001397
7	Ebola Virus Pathway on Host WP4217	0.001455
8	SARS-CoV-2 innate immunity evasion and cell-specific immune response WP5039	0.001682
9	Allograft Rejection WP2328	0.003156
10	Toll-like Receptor Signaling related to MyD88 WP3858	0.003584

Figure S2

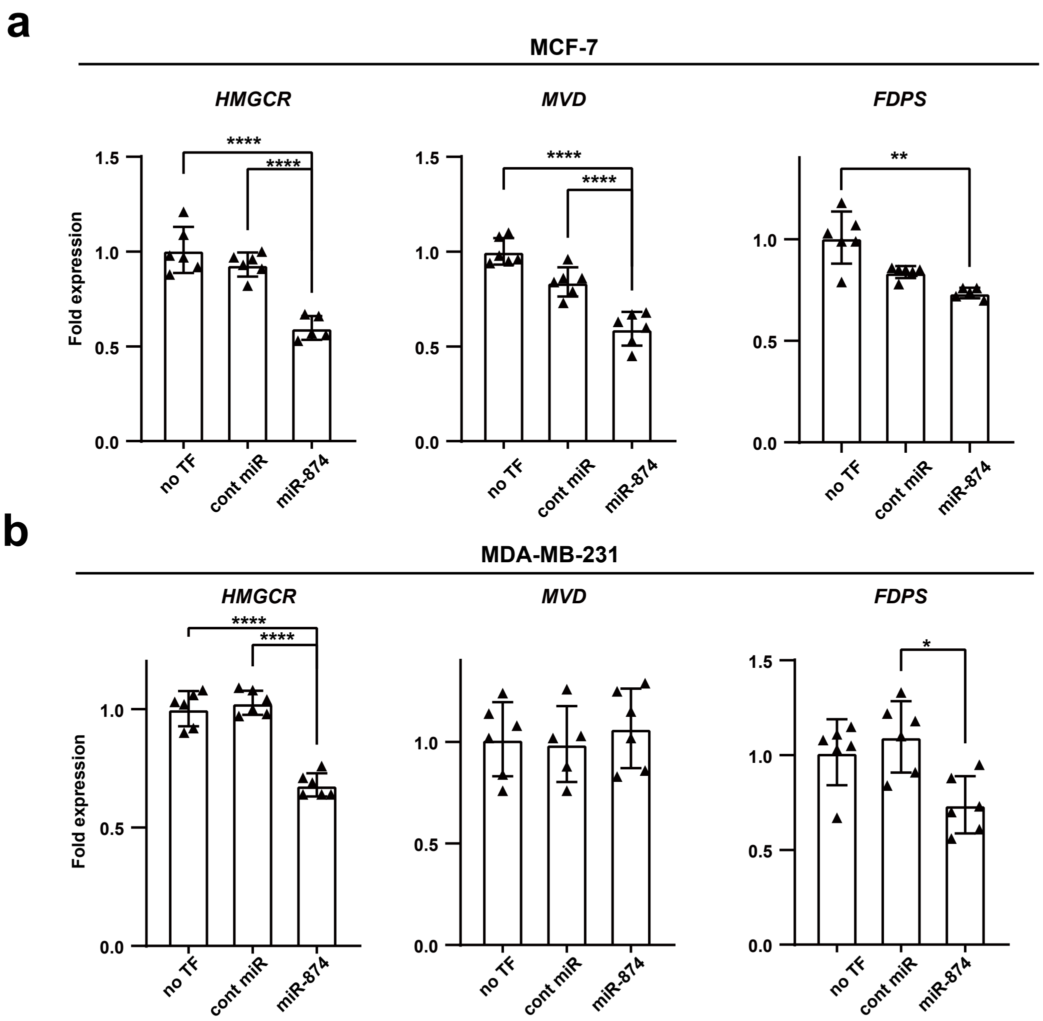
a
MCF-7
HMGCR
MVD
FDPS
Fold expression
1.5
1.0
0.5
0.0
****
****
**
no TF
cont miR
miR-874
b
MDA-MB-231
*

Figure S3

Experiment 1

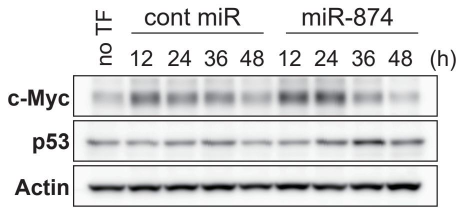

Experiment 2

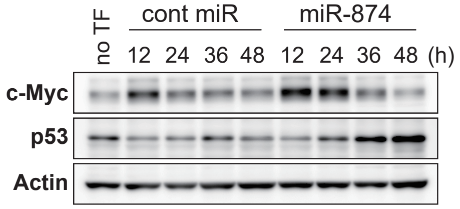

Experiment 3

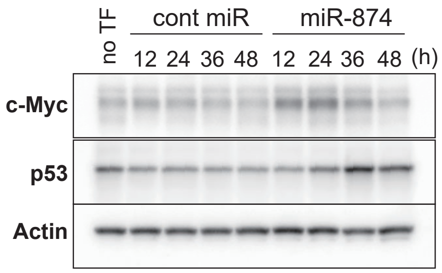

Experiment 4

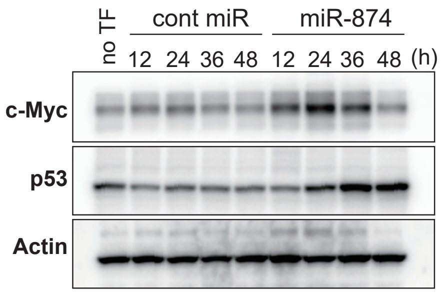

Figure S4

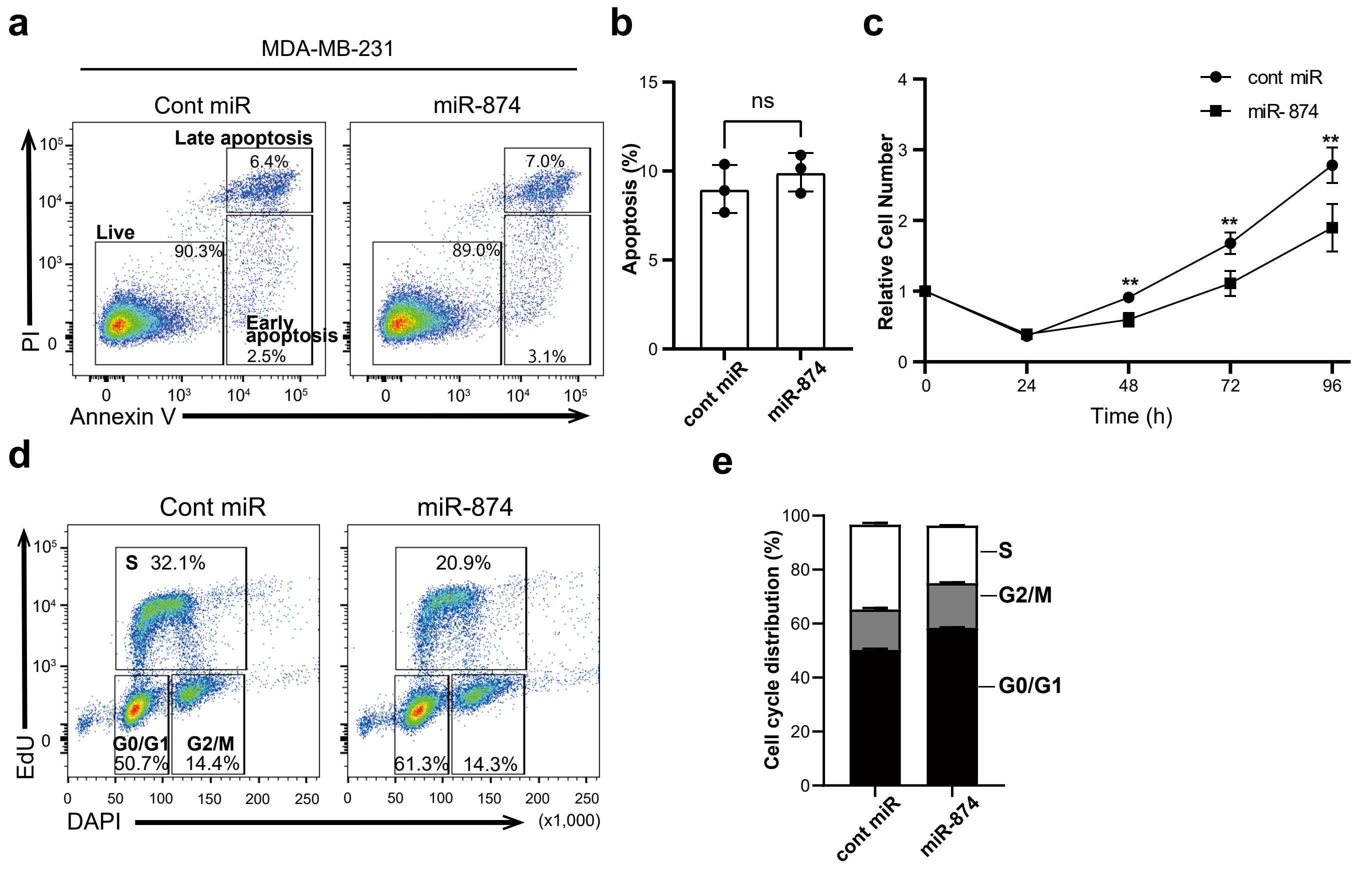

a
MDA-MB-231
Cont miR
miR-874
Late apoptosis
6.4%
7.0%
Live
90.3%
89.0%
Early apoptosis
2.5%
3.1%
PI
Annexin V
b
ns
Apoptosis (%)
cont miR
miR-874
c
cont miR
miR- 874
Relative Cell Number
Time (h)
**
d
Cont miR
miR-874
S 32.1%
20.9%
G0/G1
50.7%
G2/M
14.4%
61.3%
14.3%
EdU
DAPI
(x1,000)
e
Cell cycle distribution (%)
S
G2/M
G0/G1
cont miR
miR-874

Figure S5

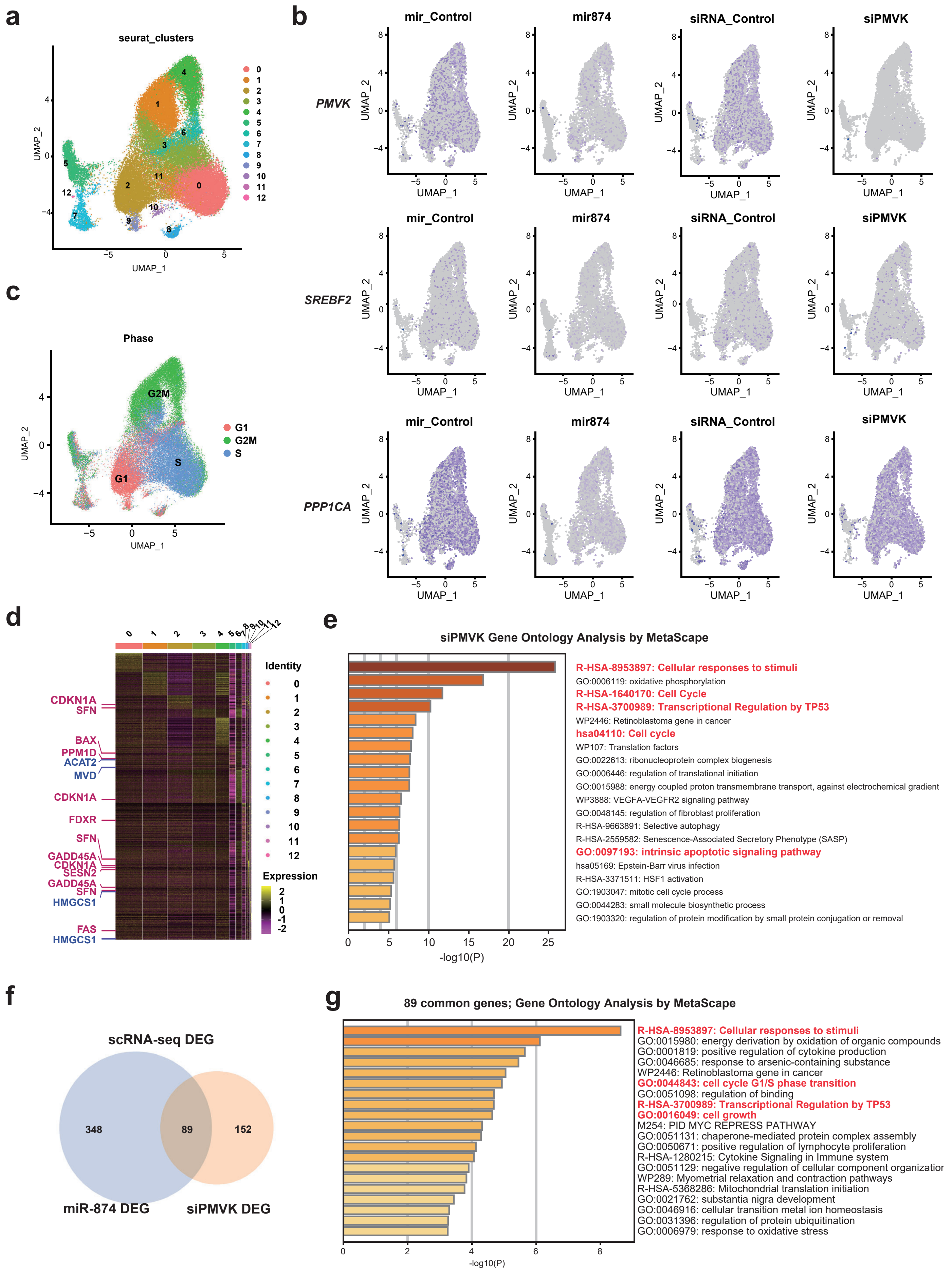
a
seurat_clusters
b
PMVK
SREBF2
PPP1CA
mir_Control
mir874
siRNA_Control
siPMVK
UMAP_1
UMAP_2
c
Phase
G1
G2M
S
d
Identity
Expression
CDKN1A
SFN
BAX
PPM1D
ACAT2
MVD
FDXR
GADD45A
SESN2
HMGCS1
FAS
e
siPMVK Gene Ontology Analysis by MetaScape
R-HSA-8953897: Cellular responses to stimuli
GO:0006119: oxidative phosphorylation
R-HSA-1640170: Cell Cycle
R-HSA-3700989: Transcriptional Regulation by TP53
WP2446: Retinoblastoma gene in cancer
hsa04110: Cell cycle
WP107: Translation factors
GO:0022613: ribonucleoprotein complex biogenesis
GO:0006446: regulation of translational initiation
GO:0015988: energy coupled proton transmembrane transport, against electrochemical gradient
WP3888: VEGFA-VEGFR2 signaling pathway
GO:0048145: regulation of fibroblast proliferation
R-HSA-9663891: Selective autophagy
R-HSA-2559582: Senescence-Associated Secretory Phenotype (SASP)
GO:0097193: intrinsic apoptotic signaling pathway
hsa05169: Epstein-Barr virus infection
R-HSA-3371511: HSF1 activation
GO:1903047: mitotic cell cycle process
GO:0044283: small molecule biosynthetic process
GO:1903320: regulation of protein modification by small protein conjugation or removal
-log10(P)
f
scRNA-seq DEG
348
89
152
miR-874 DEG
siPMVK DEG
g
89 common genes; Gene Ontology Analysis by MetaScape
R-HSA-8953897: Cellular responses to stimuli
GO:0015980: energy derivation by oxidation of organic compounds
GO:0001819: positive regulation of cytokine production
GO:0046685: response to arsenic-containing substance
WP2446: Retinoblastoma gene in cancer
GO:0044843: cell cycle G1/S phase transition
GO:0051098: regulation of binding
R-HSA-3700989: Transcriptional Regulation by TP53
GO:0016049: cell growth
M254: PID MYC REPRESS PATHWAY
GO:0051131: chaperone-mediated protein complex assembly
GO:0050671: positive regulation of lymphocyte proliferation
R-HSA-1280215: Cytokine Signaling in Immune system
GO:0051129: negative regulation of cellular component organizatior
WP289: Myometrial relaxation and contraction pathways
R-HSA-5368286: Mitochondrial translation initiation
GO:0021762: substantia nigra development
GO:0046916: cellular transition metal ion homeostasis
GO:0031396: regulation of protein ubiquitination
GO:0006979: response to oxidative stress
-log10(P)

Figure S6

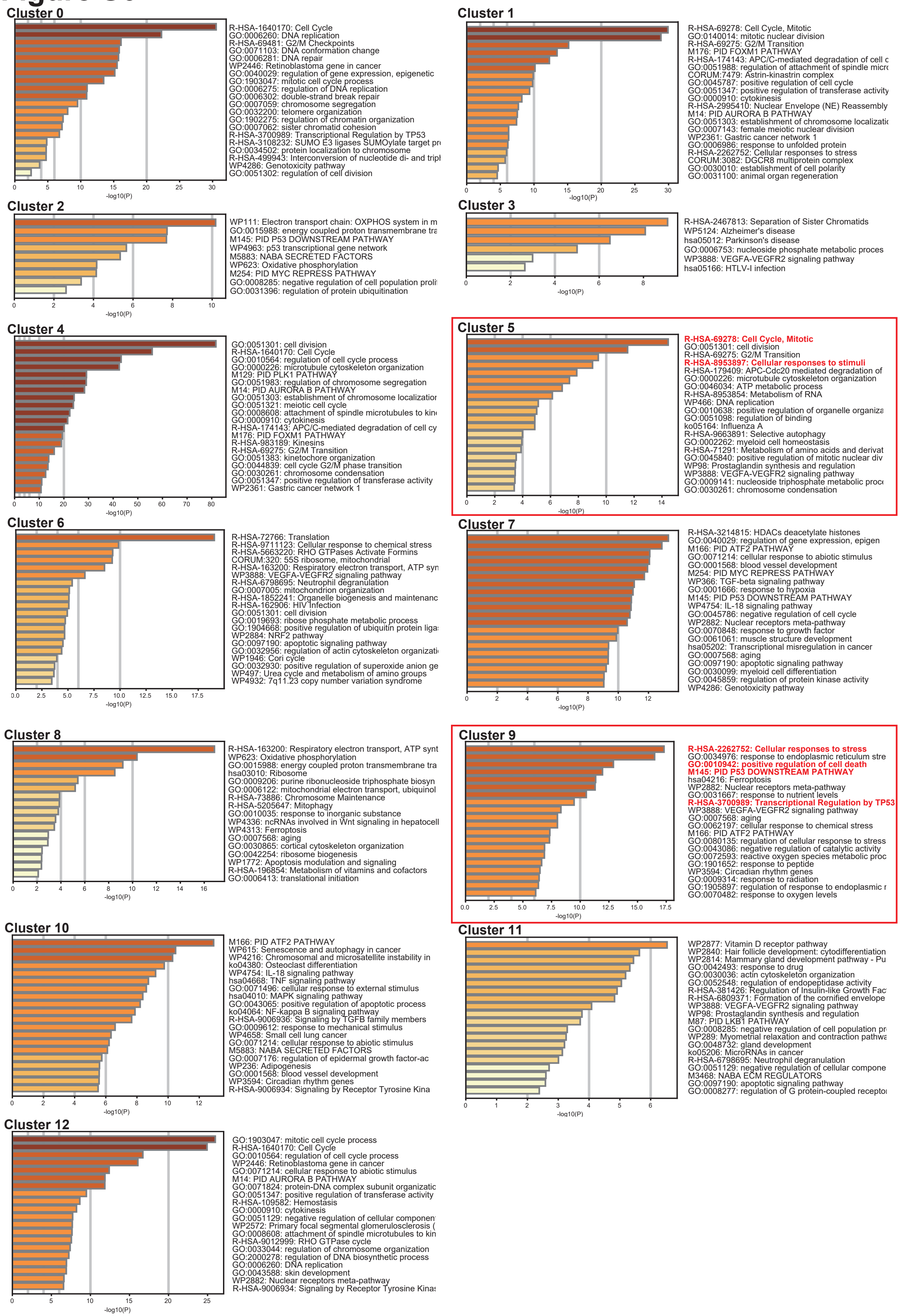
Cluster 0
R-HSA-1640170: Cell Cycle
GO:0006260: DNA replication
R-HSA-69481: G2/M Checkpoints
GO:0071103: DNA conformation change
GO:0006281: DNA repair
WP2446: Retinoblastoma gene in cancer
GO:0040029: regulation of gene expression, epigenetic
GO:1903047: mitotic cell cycle process
GO:0006275: regulation of DNA replication
GO:0006302: double-strand break repair
GO:0007059: chromosome segregation
GO:0032200: telomere organization
GO:1902275: regulation of chromatin organization
GO:0007062: sister chromatid cohesion
R-HSA-3700989: Transcriptional Regulation by TP53
R-HSA-3108232: SUMO E3 ligases SUMOylate target pr
GO:0034502: protein localization to chromosome
R-HSA-499943: Interconversion of nucleotide di- and tripl
WP4286: Genotoxicity pathway
GO:0051302: regulation of cell division
-log10(P)
Cluster 1
R-HSA-69278: Cell Cycle, Mitotic
GO:0140014: mitotic nuclear division
R-HSA-69275: G2/M Transition
M176: PID FOXM1 PATHWAY
R-HSA-174143: APC/C-mediated degradation of cell c
GO:0051988: regulation of attachment of spindle micro
CORUM:7479: Astrin-kinastrin complex
GO:0045787: positive regulation of cell cycle
GO:0051347: positive regulation of transferase activity
GO:0000910: cytokinesis
R-HSA-2995410: Nuclear Envelope (NE) Reassembly
M14: PID AURORA B PATHWAY
GO:0051303: establishment of chromosome localizatio
GO:0007143: female meiotic nuclear division
WP2361: Gastric cancer network 1
GO:0006986: response to unfolded protein
R-HSA-2262752: Cellular responses to stress
CORUM:3082: DGCR8 multiprotein complex
GO:0030010: establishment of cell polarity
GO:0031100: animal organ regeneration
-log10(P)
Cluster 2
WP111: Electron transport chain: OXPHOS system in m
GO:0015988: energy coupled proton transmembrane tra
M145: PID P53 DOWNSTREAM PATHWAY
WP4963: p53 transcriptional gene network
M5883: NABA SECRETED FACTORS
WP623: Oxidative phosphorylation
M254: PID MYC REPRESS PATHWAY
GO:0008285: negative regulation of cell population proli
GO:0031396: regulation of protein ubiquitination
-log10(P)
Cluster 3
R-HSA-2467813: Separation of Sister Chromatids
WP5124: Alzheimer's disease
hsa05012: Parkinson's disease
GO:0006753: nucleoside phosphate metabolic proces
WP3888: VEGFA-VEGFR2 signaling pathway
hsa05166: HTLV-I infection
-log10(P)
Cluster 4
GO:0051301: cell division
R-HSA-1640170: Cell Cycle
GO:0010564: regulation of cell cycle process
GO:0000226: microtubule cytoskeleton organization
M129: PID PLK1 PATHWAY
GO:0051983: regulation of chromosome segregation
M14: PID AURORA B PATHWAY
GO:0051303: establishment of chromosome localizatior
GO:0051321: meiotic cell cycle
GO:0008608: attachment of spindle microtubules to kin
GO:0000910: cytokinesis
R-HSA-174143: APC/C-mediated degradation of cell cy
M176: PID FOXM1 PATHWAY
R-HSA-983189: Kinesins
R-HSA-69275: G2/M Transition
GO:0051383: kinetochore organization
GO:0044839: cell cycle G2/M phase transition
GO:0030261: chromosome condensation
GO:0051347: positive regulation of transferase activity
WP2361: Gastric cancer network 1
-log10(P)
Cluster 5
R-HSA-69278: Cell Cycle, Mitotic
GO:0051301: cell division
R-HSA-69275: G2/M Transition
R-HSA-8953897: Cellular responses to stimuli
R-HSA-179409: APC-Cdc20 mediated degradation of
GO:0000226: microtubule cytoskeleton organization
GO:0046034: ATP metabolic process
R-HSA-8953854: Metabolism of RNA
WP466: DNA replication
GO:0010638: positive regulation of organelle organiza
GO:0051098: regulation of binding
ko05164: Influenza A
R-HSA-9663891: Selective autophagy
GO:0002262: myeloid cell homeostasis
R-HSA-71291: Metabolism of amino acids and derivat
GO:0045840: positive regulation of mitotic nuclear div
WP98: Prostaglandin synthesis and regulation
WP3888: VEGFA-VEGFR2 signaling pathway
GO:0009141: nucleoside triphosphate metabolic proce
GO:0030261: chromosome condensation
-log10(P)
Cluster 6
R-HSA-72766: Translation
R-HSA-9711123: Cellular response to chemical stress
R-HSA-5663220: RHO GTPases Activate Formins
CORUM:320: 55S ribosome, mitochondrial
R-HSA-163200: Respiratory electron transport, ATP syn
WP3888: VEGFA-VEGFR2 signaling pathway
R-HSA-6798695: Neutrophil degranulation
GO:0007005: mitochondrion organization
R-HSA-1852241: Organelle biogenesis and maintenanc
R-HSA-162906: HIV Infection
GO:0051301: cell division
GO:0019693: ribose phosphate metabolic process
GO:1904668: positive regulation of ubiquitin protein liga
WP2884: NRF2 pathway
GO:0097190: apoptotic signaling pathway
GO:0032956: regulation of actin cytoskeleton organizati
WP1946: Cori cycle
GO:0032930: positive regulation of superoxide anion ge
WP497: Urea cycle and metabolism of amino groups
WP4932: 7q11.23 copy number variation syndrome
-log10(P)
Cluster 7
R-HSA-3214815: HDACs deacetylate histones
GO:0040029: regulation of gene expression, epigen
M166: PID ATF2 PATHWAY
GO:0071214: cellular response to abiotic stimulus
GO:0001568: blood vessel development
M254: PID MYC REPRESS PATHWAY
WP366: TGF-beta signaling pathway
GO:0001666: response to hypoxia
M145: PID P53 DOWNSTREAM PATHWAY
WP4754: IL-18 signaling pathway
GO:0045786: negative regulation of cell cycle
WP2882: Nuclear receptors meta-pathway
GO:0070848: response to growth factor
GO:0061061: muscle structure development
hsa05202: Transcriptional misregulation in cancer
GO:0007568: aging
GO:0097190: apoptotic signaling pathway
GO:0030099: myeloid cell differentiation
GO:0045859: regulation of protein kinase activity
WP4286: Genotoxicity pathway
-log10(P)
Cluster 8
R-HSA-163200: Respiratory electron transport, ATP synt
WP623: Oxidative phosphorylation
GO:0015988: energy coupled proton transmembrane tra
hsa03010: Ribosome
GO:0009206: purine ribonucleoside triphosphate biosyn
GO:0006122: mitochondrial electron transport, ubiquinol
R-HSA-73886: Chromosome Maintenance
R-HSA-5205647: Mitophagy
GO:0010035: response to inorganic substance
WP4336: ncRNAs involved in Wnt signaling in hepatocell
WP4313: Ferroptosis
GO:0007568: aging
GO:0030865: cortical cytoskeleton organization
GO:0042254: ribosome biogenesis
WP1772: Apoptosis modulation and signaling
R-HSA-196854: Metabolism of vitamins and cofactors
GO:0006413: translational initiation
-log10(P)
Cluster 9
R-HSA-2262752: Cellular responses to stress
GO:0034976: response to endoplasmic reticulum stre
GO:0010942: positive regulation of cell death
M145: PID P53 DOWNSTREAM PATHWAY
hsa04216: Ferroptosis
WP2882: Nuclear receptors meta-pathway
GO:0031667: response to nutrient levels
R-HSA-3700989: Transcriptional Regulation by TP53
WP3888: VEGFA-VEGFR2 signaling pathway
GO:0007568: aging
GO:0062197: cellular response to chemical stress
M166: PID ATF2 PATHWAY
GO:0080135: regulation of cellular response to stress
GO:0043086: negative regulation of catalytic activity
GO:0072593: reactive oxygen species metabolic proc
GO:1901652: response to peptide
WP3594: Circadian rhythm genes
GO:0009314: response to radiation
GO:1905897: regulation of response to endoplasmic r
GO:0070482: response to oxygen levels
-log10(P)
Cluster 10
M166: PID ATF2 PATHWAY
WP615: Senescence and autophagy in cancer
WP4216: Chromosomal and microsatellite instability in
ko04380: Osteoclast differentiation
WP4754: IL-18 signaling pathway
hsa04668: TNF signaling pathway
GO:0071496: cellular response to external stimulus
hsa04010: MAPK signaling pathway
GO:0043065: positive regulation of apoptotic process
ko04064: NF-kappa B signaling pathway
R-HSA-9006936: Signaling by TGFB family members
GO:0009612: response to mechanical stimulus
WP4658: Small cell lung cancer
GO:0071214: cellular response to abiotic stimulus
M5883: NABA SECRETED FACTORS
GO:0007176: regulation of epidermal growth factor-ac
WP236: Adipogenesis
GO:0001568: blood vessel development
WP3594: Circadian rhythm genes
R-HSA-9006934: Signaling by Receptor Tyrosine Kina
-log10(P)
Cluster 11
WP2877: Vitamin D receptor pathway
WP2840: Hair follicle development: cytodifferentiation
WP2814: Mammary gland development pathway - Pu
GO:0042493: response to drug
GO:0030036: actin cytoskeleton organization
GO:0052548: regulation of endopeptidase activity
R-HSA-381426: Regulation of Insulin-like Growth Fac
R-HSA-6809371: Formation of the cornified envelope
WP3888: VEGFA-VEGFR2 signaling pathway
WP98: Prostaglandin synthesis and regulation
M87: PID LKB1 PATHWAY
GO:0008285: negative regulation of cell population pr
WP289: Myometrial relaxation and contraction pathwa
GO:0048732: gland development
ko05206: MicroRNAs in cancer
R-HSA-6798695: Neutrophil degranulation
GO:0051129: negative regulation of cellular compone
M3468: NABA ECM REGULATORS
GO:0097190: apoptotic signaling pathway
GO:0008277: regulation of G protein-coupled recepto
-log10(P)
Cluster 12
GO:1903047: mitotic cell cycle process
R-HSA-1640170: Cell Cycle
GO:0010564: regulation of cell cycle process
WP2446: Retinoblastoma gene in cancer
GO:0071214: cellular response to abiotic stimulus
M14: PID AURORA B PATHWAY
GO:0071824: protein-DNA complex subunit organizatic
GO:0051347: positive regulation of transferase activity
R-HSA-109582: Hemostasis
GO:0000910: cytokinesis
GO:0051129: negative regulation of cellular componen
WP2572: Primary focal segmental glomerulosclerosis (
GO:0008608: attachment of spindle microtubules to kin
R-HSA-9012999: RHO GTPase cycle
GO:0033044: regulation of chromosome organization
GO:2000278: regulation of DNA biosynthetic process
GO:0006260: DNA replication
GO:0043588: skin development
WP2882: Nuclear receptors meta-pathway
R-HSA-9006934: Signaling by Receptor Tyrosine Kina
-log10(P)

Figure S7

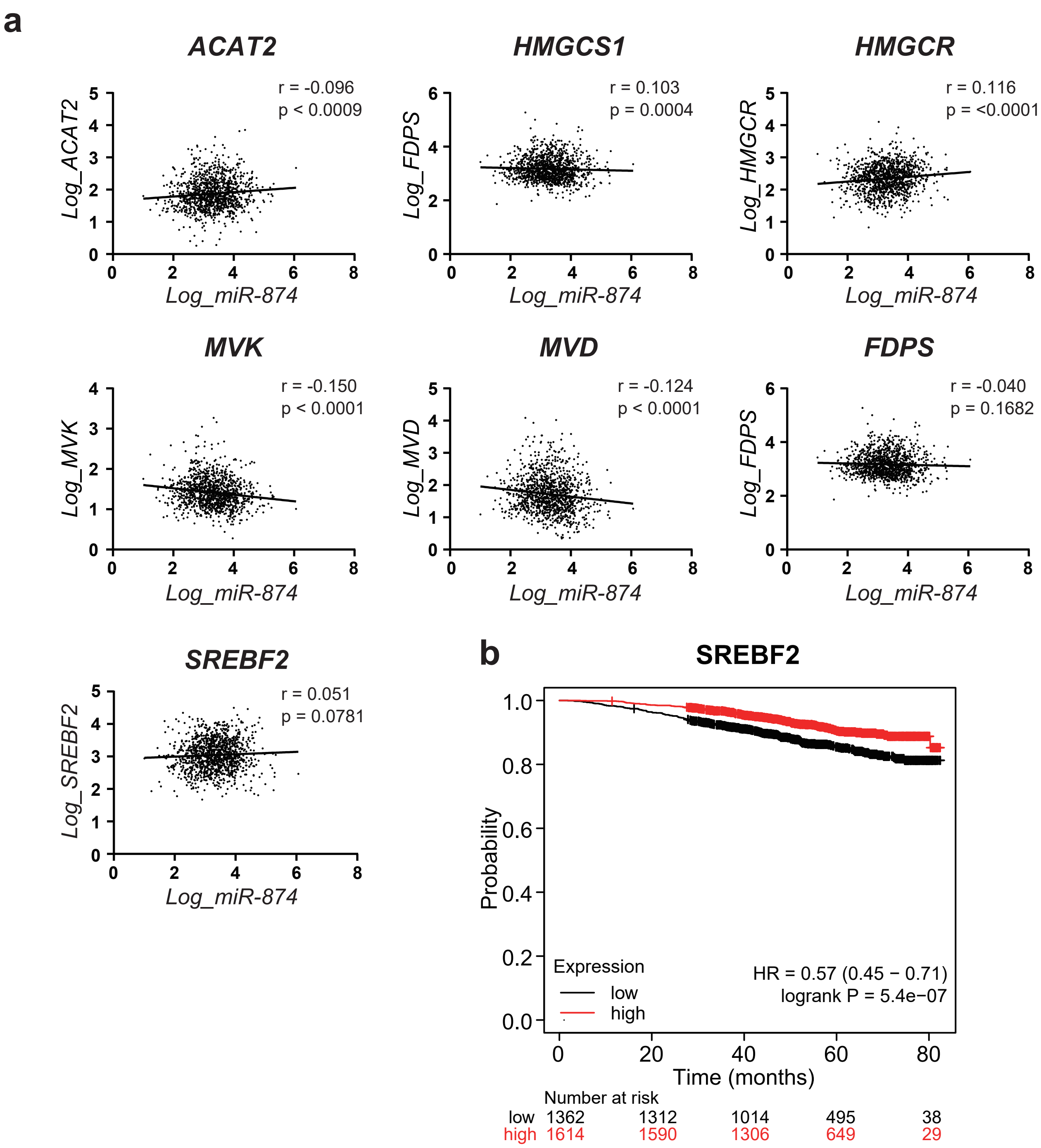
a
ACAT2
r = -0.096
p < 0.0009
Log_ACAT2
Log_miR-874
HMGCS1
r = 0.103
p = 0.0004
Log_FDPS
Log_miR-874
HMGCR
r = 0.116
p = <0.0001
Log_HMGCR
Log_miR-874
MVK
r = -0.150
p < 0.0001
Log_MVK
Log_miR-874
MVD
r = -0.124
p < 0.0001
Log_MVD
Log_miR-874
FDPS
r = -0.040
p = 0.1682
Log_FDPS
Log_miR-874
SREBF2
r = 0.051
p = 0.0781
Log_SREBF2
Log_miR-874
b
SREBF2
Probability
Expression
low
high
HR = 0.57 (0.45 − 0.71)
logrank P = 5.4e−07
Time (months)
Number at risk
low 1362 1312 1014 495 38
high 1614 1590 1306 649 29
